# Air Pollution and Alzheimer’s Disease: A Systematic Review and Meta-Analysis

**DOI:** 10.3390/jcm15114163

**Published:** 2026-05-28

**Authors:** Ludovico Baiamonte, Domenico Tarantino, Manuela Lodico, Giovanna Bellante, Claudia Migliazzo, Patrizio Allegra, Laura Maniscalco, Tommaso Piccoli, Nicola Vanacore, Domenica Matranga, Giuseppe Salemi

**Affiliations:** 1 Department of Diagnostic, Interventional and Stroke Radiology, UOC Neurology, AOUP ‘P. Giaccone’, 90100 Palermo, Italy; ludovico.baiamonte@community.unipa.it (L.B.); tarantino.domenico95@gmail.com (D.T.); giovanna.bellante01@you.unipa.it (G.B.); claudia.migliazzo@community.unipa.it (C.M.); tommaso.piccoli@unipa.it (T.P.); giuseppe.salemi@unipa.it (G.S.); 2 Department of Biomedicine, Neuroscience and Advanced Diagnostics, University of Palermo, 90100 Palermo, Italy; 3 Department of Health Promotion, Mother and Child Care, Internal Medicine and Medical Specialties, University of Palermo, Via del Vespro 129, 90100 Palermo, Italy; laura.maniscalco04@unipa.it (L.M.); domenica.matranga@unipa.it (D.M.); 4 National Center for Disease Prevention and Health Promotion, Italian National Institute of Health, 00100 Rome, Italy; nicola.vanacore@iss.it

**Keywords:** Alzheimer’s disease, air pollution, systematic review, risk factors, epidemiology

## Abstract

**Background/Objectives:** Alzheimer’s disease (AD) is the most common cause of dementia. Among the various factors associated with the risk of AD, growing attention in recent years has focused on environmental influences, particularly air pollution. The association between air pollutants and AD remains inconclusive due to heterogeneity in available studies. Given these gaps, we performed a systematic review on the topic. **Methods:** We systematically searched Pubmed, Embase and Scopus. Retrieved records underwent screening by title and abstract and then in full text. We included studies quantitatively exploring the association between exposure to air pollutants and risk of AD. We performed a meta-analysis to identify a pooled estimate of the impact of each pollutant on the probability of developing AD. **Results:** We retrieved 1081 records and included 27 studies. We found a significant association between PM_2.5_ levels and AD risk (HR 1.74, 95%CI 1.36–2.23). Our data did not support a relevant role for the other pollutants we analyzed (for PM_10_ HR = 1.35, 95%CI: 0.86–2.11; for NO_2_ HR = 1.34, 95%CI 0.96–1.86; and for O_3_ HR = 1.03, 95%CI 0.68–1.57). **Conclusions:** PM_2.5_ emerged as the pollutant most strongly and consistently associated with an increased risk of AD. This robust and statistically significant association underscores the potential neurotoxic effects of fine particulate matter. For other pollutants, a clear role was not found. These results should be interpreted with caution, due to high heterogeneity in the definition of AD in the included studies (in most cases, a clinical definition was used). More research will be needed in the future.

## 1. Introduction

Dementia is a rapidly growing condition, with a new case diagnosed every four seconds and prevalence doubling every twenty years [1]. Between 1990 and 2016, global cases increased from 20.2 million to 43.8 million, while dementia-related deaths more than doubled, reaching 1.62 million in 2019 and ranking as the seventh leading cause of death worldwide [2]. Global medical costs of dementia are estimated up to US $213 billion, and the burden rises to US $ 1313 billion when considering indirect costs [3].

Among the heterogeneous causes of dementia, Alzheimer’s disease (AD) is the most common one, accounting for an estimated 60 to 80% of cases [3]. AD is a neurodegenerative disorder that is marked clinically by a decline in episodic memory and other cognitive domains, with subsequent impact on autonomy in daily living activities, and pathologically by extracellular plaques of amyloid-beta (Aβ) and intracellular aggregates of hyperphosphorylated tau protein [4]. Diagnostic criteria for AD have undergone a progressive shift from the clinical dimension, as in those proposed by the National Institute of Neurological and Communicative Disorders and Stroke (NINCS) and the Alzheimer’s Disease and Related Disorders Association (ADRDAs) [5], to a purely biological or clinical–biological dimension, as in those proposed by the National Institute on Aging and the Alzheimer’s Association (NIA-AAs) [6] or those of the International Working Group (IWG) [7]. Biological diagnosis of AD is based on the detection of imaging or biochemical biomarkers of Aβ proteinopathy and of deposition of hyperphosphorylated tau, with biomarkers of neuronal injury or degeneration as adjunctive information (ATN system).

Given the projected increase in AD cases due to the aging of population and the continued absence of a cure (even if anti-β amyloid monoclonal antibodies and noninvasive brain stimulation could provide new opportunities in this field [8,9]), there is growing interest in identifying modifiable environmental risk factors for this disease [2]. Among the various factors associated with the risk of dementia in general and AD in particular—including age, sex, education level, presence of the APOE4 allele, metabolic syndrome and lifestyle—growing attention in recent years has focused on environmental influences, particularly air pollution [1]. In 2020, the Lancet Commission formally recognized air pollution as a modifiable risk factor for senile dementia, sparking intense research into the effects of various pollutants on cognitive function [10]. The Environmental Protection Agency (EPA) has prioritized causal studies on this topic, acknowledging that pollution is a strategic target for public health policies [11].

However, despite the growing body of epidemiological evidence, the association between air pollution and dementia remains inconclusive due to heterogeneity in study designs, populations examined, exposure assessment methods, and clinical outcome definitions. Understanding the biological mechanisms involved, particularly the impact of pollutants on the metabolic activity of AD-related pathological proteins, is limited by the scarcity of clinical data [10]. Some progress has been observed in recent years, but in most cases the focus has been on dementia from any causes and not on AD in particular, as shown by a recent review on this topic [12].

Considering these gaps, the present study aims to conduct a systematic review and meta-analysis of the available literature to critically and quantitatively assess the association between exposure to air pollutants and the risk of AD. The goal is to provide more robust and integrated evidence to inform future public health strategies and primary prevention policies.

## 2. Methods

We performed a systematic review and meta-analysis and reported our findings according to the Preferred Reporting Items for Systematic Reviews and Meta-Analyses (PRISMAs) guidelines [13,14]. For the PRISMA checklist, see Table S1 in Supplementary Materials. We registered our review on the International Prospective Register of Systematic Reviews (PROSPERO; CRD420251059361).

### 2.1. Data Sources and Search Strategy

We systematically searched MEDLINE (via PubMed), Embase (via Ovid) and Scopus databases from inception to 16 May 2025. Polyglot tool [15], provided by the systematic review accelerator (SRA) [16], was used to assist us in string conversion between different sources. The search strings are included in Tables S2–S4 in Supplementary Materials. The reference list of studies selected for inclusion and published systematic reviews on the same topic were also screened for studies that met our inclusion criteria. Following search, retrieved titles were collated in Zotero software (version 6.0.36); then, duplicated items were removed using the Deduplicator tool [17] of the SRA. Screening by title and abstract was performed by two reviewers (DT and ML) using Rayyan platform [18]; disagreements were solved by discussion between the two authors and, if needed, through involvement of a third one. The same method was used for full-text screening.

### 2.2. Eligibility Criteria

We included studies (1) adopting a cohort or case–control design (other study designs were excluded due to their higher susceptibility to bias); (2) quantitatively exploring the association between exposure to air pollutants and AD through the use of adequate effect measures; (3) considering an exposure period of one year or more (in order to ensure a minimum duration of exposure and in accordance with previous reviews on the same topic [12]); (4) defining AD according to clinical and/or biomarker-based diagnosis; (5) including at least five exposed participants in each group; (6) published in peer-reviewed journals; and (7) written in English language. A broad type of air pollutants was considered, including, but not limited to, particulate matter < 2.5 μm in diameter (fine particulate matter, PM_2.5_), particulate matter < 10 μm in diameter (coarse particulate matter, PM_10_), ozone (O_3_), nitrogen dioxide (NO_2_), sulfur dioxide (SO_2_), and carbon monoxide (CO).

We excluded: (1) book chapters, reviews, letters, case reports or conference abstracts; (2) studies not providing enough data to calculate the risk estimates; (3) studies where exposure to air pollutants was not quantitatively measured; (4) studies where dementia was not clearly defined as AD; and (5) studies on animal models.

### 2.3. Data Extraction

Data extraction was performed using an electronic sheet. Extracted data for each study regarded publication year, region, study design, sample size, mean or median age of participants overall and in each group, gender of participants overall and in each group, type of air pollutants, unit of exposure, AD ascertainment method, age at diagnosis, effect size and its variance, and covariate adjustment. Measures of association were recorded with 95% confidence intervals, unit of exposure (μg/m^3^, ppb, etc.) and scaling factor (e.g., 1 μg/m^3^, 5 μg/m^3^, and 10 μg/m^3^).

### 2.4. Outcomes

The main outcome was the identification of a pooled estimate of the effect measures regarding the association between the exposure to air pollutants and the risk of developing AD (for each type of air pollutant separately). Secondary outcomes included evaluation of the risk of bias of included studies and exploration of possible sources of heterogeneity.

### 2.5. Risk of Bias Assessment

Assessment of risk of bias in included studies was performed using the same methods adopted in previous reviews about the same topic [19]; in particular, the quality appraisal was focused on the source of data, the design of the study, the information on participants provided by each study and the approach to sources of heterogeneity and to missing data. Publication bias was assessed using a funnel plot with Egger’s statistics.

### 2.6. Data Synthesis and Analysis

For each type of air pollutant, effect measures of association with the risk of AD were log-transformed and pooled using a random-effect model with Hartung–Knapp–Sidik–Jonkman adjustment. Before being entered into the analysis, effect sizes were converted to express the AD risk variation for a standard increase in the pollutant (a value of 10 μg/m^3^ was chosen as the standard one); to do this, the natural logarithm of the hazard ratio (logHR) was multiplied by the ratio between 10 and the original exposure concentration. When the original exposure was expressed in parts per billion (ppb), it was converted to μg/m^3^ through multiplication by the ratio between the pollutant’s molecular weight and the molar volume (a standard molar volume of 24.45 L/mol was used).

Two-sided *p* values lower than 0.05 were considered statistically significant unless otherwise stated, and the individual and pooled effect sizes were shown in a forest plot. The statistical heterogeneity among the studies was assessed by the Cochran’s Q statistic (*p* values < 0.10 were considered indicative of statistically significant heterogeneity) and I^2^ statistic (values less than 25% represent mild heterogeneity, values between 25% and 50% represent moderate heterogeneity, and values greater than 50% represent large heterogeneity).

To address the problem of multiple studies based on the same database, sensitivity analyses were run with the exclusion of overlapping articles.

## 3. Results

### 3.1. Study Selection

**Figure 1:**
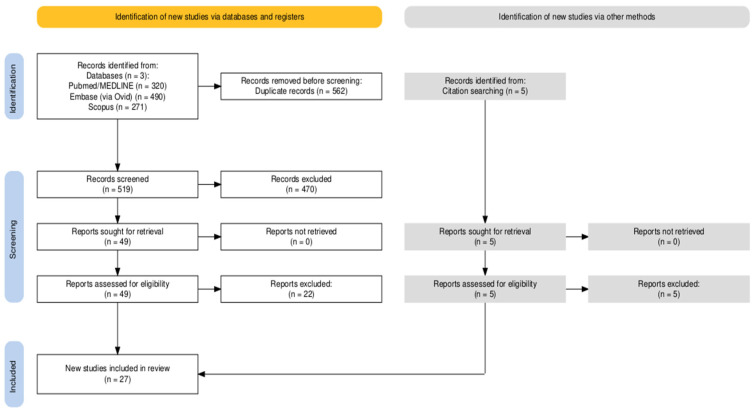
PRISMA flowchart for the study selection process.

Our search retrieved a total of 1081 records. Among them, 562 duplicates were identified and excluded; the remaining 519 titles and abstracts were screened for relevance and 49 of them were deemed eligible for inclusion in full-text review. Moreover, five records were identified through manual search in reference lists of the included articles and of relevant reviews on the same topic; all of them were retrieved in full text and were included in the following step of the selection process. A total of 54 studies underwent full-text review and 27 of them [1,2,10,11,20,21,22,23,24,25,26,27,28,29,30,31,32,33,34,35,36,37,38,39,40,41,42] met eligibility criteria and were included in the final review. A detailed flowchart of the study selection process according to PRISMA guidelines can be found in Figure 1. Table S5 in Supplementary Materials lists the studies excluded in full-text review [43,44,45,46,47,48,49,50,51,52,53,54,55,56,57,58,59,60,61,62,63,64,65,66,67,68,69] and the reason for their exclusion.

### 3.2. Characteristics of Included Studies

We included 27 studies in our review [1,2,10,11,20,21,22,23,24,25,26,27,28,29,30,31,32,33,34,35,36,37,38,39,40,41,42]; detailed characteristics for each study are listed in Table 1. Publication years ranged from 2015 to 2025; eight studies were conducted in America, thirteen in Europe, and six in Asia. Two studies [11,31] adopted a retrospective cohort design, and only one [1] a case–control approach.

Data on exposure to air pollutants were collected ad hoc only for one study [41]; the other ones used data from environmental monitoring systems, both ground- and satellite-based. Twenty-five studies considered the exposure to PM_2.5_ (Table 2), seven to PM_10_ (Table 3), twelve to NO_2_ (Table 4) and six to O_3_ (Table 5). No data were found regarding CO and SO_2_. Seventeen studies [1,2,11,20,22,23,24,27,29,30,32,33,34,36,38,40,41] considered the exposure to pollutants during a follow-up of 10 years or more, seven [10,25,26,31,37,39,42] for 5–10 years, and three [21,28,35] for less than 5 years.

With regard to data on AD cases, seven studies [11,21,27,32,33,40,42] used health insurance datasets, 14 [2,10,20,22,23,24,25,26,30,31,34,36,37,39] used data from general health monitoring systems, four [10,29,38,41] used dementia epidemiological databases, two [1,36] used hospital records, one [35] data from a drug prescription monitoring system and one [24] ad hoc designed questionnaires. Only one study [10] used a biological definition of AD (NIA-AA 2011 criteria), at least in one of its subcohorts. Among studies using clinical and/or neuropsychological definitions, two [1,42] adopted the NINCS-ADRDA criteria, whereas other two [38,41] those provided by the Diagnostic and Statistical Manual (DSM) IV edition (in this case, a radiological evaluation was also performed). Four studies [21,24,27,32] did not provide precise information on the criteria used, while the remaining studies relied on a definition of AD based on the International Classification of Disease (ICD) codes attributed to participants by the involved centers. Two studies [34,35] considered the prescription of AD-related drugs as an alternative criterion for AD diagnosis. Even if not explicitly stated, four studies [10,28,38,41] seem to also include subjects with mild cognitive impairment and two of them [38,41] considered a worsening neuropsychological profile as a valid outcome for AD diagnosis.

### 3.3. Quality Appraisal

The results of quality evaluation of included studies are listed in Table S6 of Supplementary Materials. The average quality score was 9.4; a score of 9–10 was observed in 22 studies [1,2,16,18,19,20,21,23,24,25,26,28,29,30,31,32,33,34,35,36,37,39], a score of 6 in one study [31], and a score of 7–8 in 4 studies [11,21,26,42].

### 3.4. Association Between PM_2.5_ and AD Risk

For the exposure to PM_2.5_, the hazard ratios (HRs) from 22 studies were included in the meta-analysis (see Table 2); the other three studies on PM_2.5_ were excluded because they provided odds ratios (ORs) [31,35] or relative risks (RRs) [39]. Individual results varied widely in effect size, with some studies reporting very strong associations (Jung 2015 [42]: HR = 7.37, 95%CI 6.22–8.73; Parra 2022 [26]: HR = 4.81, 95%CI 1.80–12.84; Kioumourtzoglou 2016 [40]: HR = 4.05, 95%CI 2.86–5.73), while others showed more modest or even protective effects (Cerza 2019 [36]: HR = 0.83, 95%CI 0.73–0.95).

**Figure 2:**
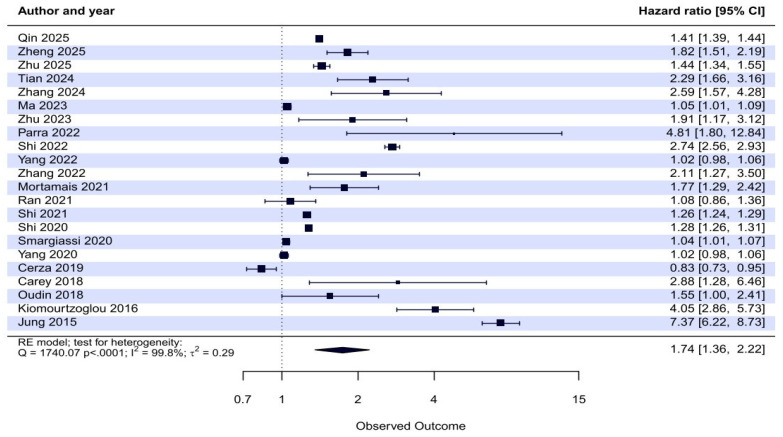
Forest plot for the meta-analysis on the association between PM_2_._5_ and AD risk [2,10,11,20,21,22,23,25,26,27,28,29,30,32,33,34,35,36,37,38,40,42].

The pooled HR was 1.74 (95%CI 1.36–2.23), indicating a statistically significant 74% increase in AD risk for an increase in PM_2.5_ concentration by 10 μg/m^3^ (Figure 2). Despite extremely high heterogeneity (I^2^ = 99.8%, τ^2^ = 0.29), the direction of the effect was consistently oriented toward increased risk in most studies. The funnel plot revealed slight asymmetry, with a greater concentration of studies on the left side of the graph, suggesting a possible publication bias (see Figure S1 in Supplementary Materials); this was confirmed by the Egger’s test (*p* = 0.008).

### 3.5. Association Between PM_10_ and AD Risk

For PM_10_ exposure, five out of six studies were included in the meta-analysis (see Table 3; the other one [1] was excluded because it provided OR instead of HR). Most individual studies indicated increased risk (Zhang 2023 [2]: HR = 1.30, 95%CI 0.95–1.79; Tian 2024 [22]: HR = 1.77, 95%CI 1.46–2.14; Zhang 2024 [23]: HR = 1.34, 95%CI 0.79–2.29), while one study suggested a possible protective effect (Cerza 2019 [36]: HR = 0.95, 95%CI 0.91–0.99). Notably, Zhu et al. [25] reported a very high HR (4.41, 95%CI 0.90–21.58), although characterized by a wide confidence interval.

**Figure 3:**
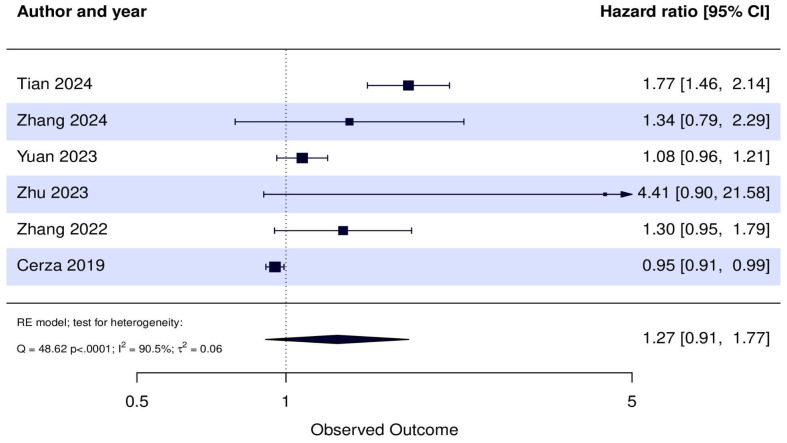
Forest plot for the meta-analysis on the association between PM_10_ and AD risk [2,22,23,24,25,36].

The meta-analysis revealed a pooled hazard ratio of 1.35 (95%CI: 0.86–2.11), suggesting a 35% increase in AD risk, although not statistically significant due to the confidence interval crossing the null value (see Figure 3 for the forest plot). Study heterogeneity was high (I^2^ = 90.5%, τ^2^ = 0.06). The funnel plot showed a relatively symmetrical distribution, suggesting a low risk of publication bias (see Figure S2 in Supplementary Materials); Egger’s test was not performed, since its reliability for meta-analyses including less than 10 studies is low.

### 3.6. Association Between NO_2_ and AD Risk

All the studies on the association between NO_2_ and AD risk provided HRs and were included in the meta-analysis (see Table 4). Individual HRs ranged from protective values (Mortamais 2021 [29]: HR = 0.88, 95%CI 0.76–1.03) to substantially elevated risks (Qin 2025 [11]: HR = 4.21, 95%CI: 3.93–4.52; Parra 2022 [26]: HR = 4.05, 95%CI: 1.43–11.42).

**Figure 4:**
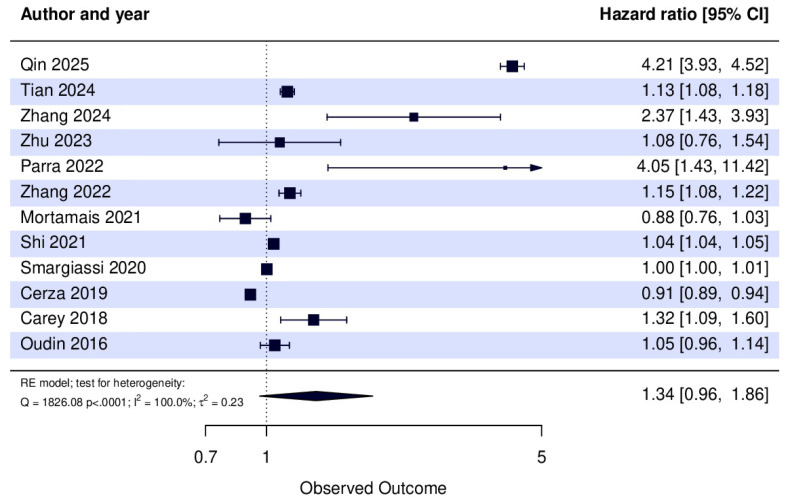
Forest plot for the meta-analysis on the association between NO_2_ and AD risk [2,11,22,23,25,26,29,32,34,36,37,41].

The pooled analysis yielded an HR of 1.34 (95%CI 0.96–1.86), thus indicating a non-statistically significant 34% increase in AD risk for the standard increase in NO_2_ concentration (Figure 4). However, the results showed extremely high heterogeneity across studies (I^2^ = 100%, τ^2^ = 0.23); the funnel plot revealed an asymmetric distribution of studies, with a lack of small studies on the left side of the graph, suggesting potential publication bias (see Figure S3 in Supplementary Materials); however, Egger’s test result was not significative (*p* = 0.071).

### 3.7. Association Between O_3_ and AD Risk

For O_3_, one study was excluded because it provided OR [1], whereas the other five were included (see Table 5). Individual results showed considerable variability as follows: some studies suggested increased risk (Jung 2015 [42]: HR = 1.68, 95%CI 1.63–1.74), while others indicated protective effects (Carey 2018 [37]: HR = 0.64, 95%CI 0.48–0.86).

**Figure 5:**
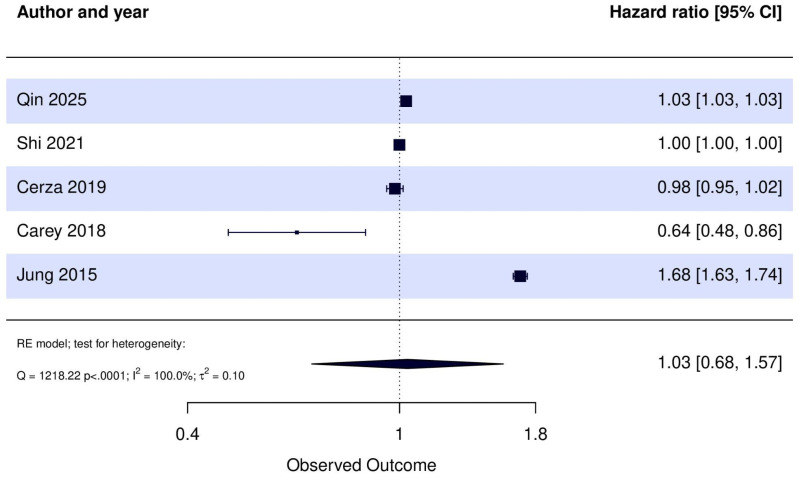
Forest plot for the meta-analysis on the association between O_3_ and AD risk [11,32,36,37,42].

The meta-analysis showed a pooled hazard ratio of 1.03 (95%CI 0.68–1.57), indicating a largely neutral effect of ozone exposure on AD risk (Figure 5). Again, heterogeneity was very high (I^2^ = 100%, τ^2^ = 0.10). See Figure S4 in Supplementary Materials for the funnel plot (Egger’s test was not performed due to the low number of studies).

### 3.8. Sensitivity Analysis

The problem of multiple studies from the same database regarded United Kingdom (UK) Biobank and United States (US) Medicare and Medicaid data. For the first database, for each pollutant we included only the study with the largest number of participants (Yuan 2023 [24] for PM_2.5_ and PM_10_, Zhang 2023 [2] for NO_2_). For Medicare data, the only study we included for each pollutant was selected applying the following criteria in sequence: (1) use of both part A and B of the database (both outpatient and inpatient data); (2) inclusion of both Medicare and Medicaid data; and (3) highest number of participants; as a result, we included the study by Zhu et al. [25] for PM_2.5_, and the one by Shi et al. [32] for the other three pollutants.

The results of our sensitivity analyses were not different from those of the main analyses: a significant association with AD risk was observed for PM_2.5_ (42,972,408 participants, HR 1.51, 95%CI 1.07–2.13), but not for PM_10_ (817,801 participants, HR 1.01, 95%CI 0.67–1.52), NO_2_ (14,788,063 participants, HR 1.03, 95%CI 0.96–1.12) or O_3_ (12,810,883 participants, HR 1.03, 95%CI 0.71–1.50). See Figures S5–S8 in Supplementary Materials for the forest plots of these analyses.

## 4. Discussion

In this systematic review and meta-analysis, we have compiled the current body of evidence regarding the link between AD and air pollution.

First, it can be observed that this topic has sparked growing interest, as shown by the increase in the number of studies on the topic, which were eight between 2015 and 2019 and 19 from 2020 on. This can probably be linked to rising concern for the effects of human activities on the environment and, by consequence, on human health. The geographical distribution of selected studies, scattered among three continents, points out the global relevance of the topic.

The main result we found was a significant influence of fine particulate matter on the risk of AD: an increase in PM_2.5_ concentration by 10 μg/m^3^ is associated with an increase in this risk by 74%. This is a relevant finding and expands existing knowledge, since previous reviews were mainly focused on the association between PM_2.5_ and dementia in general, but not on AD in particular [19,70]. Both these studies, which used different inclusion criteria and thus included different sets of articles, did not find a statistically significant result, although a tendency towards a positive association was observed; in one of them, the value of HR for air pollution was 1.95, which is not far from the one we got for AD. Apart from the pooled effect, it is worth noting that individual studies reported a wide range of HRs, which can be probably attributed to heterogeneity in study designs, inclusion criteria and specific definition of exposure (mean exposure over years, exposure trend over years or baseline exposure); also, the choice of confounding factors included as covariates in the multivariable survival analyses contributed to heterogeneity (see Table 2 for the covariates used in each study). The paper by Jung et al. [42] deserves particular attention, since it is the one with the highest point estimate of HR among included studies; this could be the result of some choices in the exposure definition, like the use of the increase in PM_2.5_ concentration over years rather than its multi-year mean and the use of an indirect estimate of PM_2.5_ concentration for the first half of the exposure period (due to the lack of direct data and the observed consistency of PM_2.5_/PM_10_ ratio over time, PM_2.5_ concentration was calculated by multiplying the known PM_10_ concentration for this fixed ratio). On the other extreme, the paper by Cerza et al. [36] is the only one with a point estimate of HR lower than 1; however, the authors themselves admit the limitations of their study, mainly caused by the uneven distribution of air pollution in Rome, the residual confounding for socioeconomic status and the differences in the use of health services.

For PM_10_, no significant association was found with AD risk. This finding is challenging, since it is not easy to understand if it is based on a real biological difference or if it is rather a result of the sample size, which was the lowest among included pollutants. It is worth noting that the existing literature does not provide relevant hints to solve this question, since one previous review included only a single article on PM_10_ [19] and another one [70] excluded studies on PM_10_ from meta-analysis. We could not demonstrate an influence of ozone concentration on the risk of AD, and this finding confirms those of previous reviews on the same subject [19,70]. The same can be said for NO_2_: also, in this case a previous review found similar results, even if the authors supposed that multiple biases in one of the articles they included (as we did too) could have influenced the result [19]. Moreover, ozone and nitrogen dioxide were the two pollutants for which the highest heterogeneity in effect sizes was observed, and this highlights the inconsistency and complexity of the findings related to these pollutants.

Given the results we found regarding the impact of air pollutants on the risk of developing AD, it is useful to provide a brief review of the possible mechanisms that drive this connection. Among them, neuroinflammation is probably the most important one. The presence of immune response and inflammation in AD pathogenesis is now widely recognized, as shown by pathological, biochemical and imaging evidence [71]; these responses cannot be considered just a consequence of degeneration, but rather a driving force in the progression of the disease, since chronic activation of microglia via proinflammatory cytokines, elicited by Aβ deposition, not only is unable to clear the plaques, but in the end increases overall amyloid toxicity through oxidative stress, synaptic neurodegeneration and other mechanisms [72]. These mechanisms are not limited to AD, but can be observed also in other neurodegenerative diseases: for example, microglial activation has been called into account to explain the neuronal toxicity of a-synuclein aggregates in Parkinson’s disease (PD) or of TAR DNA-binding protein 43 (TDP-43) in amyotrophic lateral sclerosis (ALS) [73].

The importance of neuroinflammation in AD pathogenesis seems to be confirmed by its role in the connection between the disease and some of its acquired risk factors. For example, this is the case of metabolic syndrome: it is thought that metabolic dysregulation, with particular reference to hyperglycemia and insulin resistance, can lead to inflammation and oxidative stress through metabolic reprogramming of microglia and vascular damage (this would explain how newer therapies for diabetes, such as inhibitors of sodium-glucose cotransporter 2, could be useful in the prevention of AD) [74,75,76]. The same can be said about air pollution, which is the topic of our review: inhaled pollutants have often been deemed responsible of enhancing inflammation and oxidative stress in the central nervous system, thus increasing the risk not only of AD, but also of dementia in general and of other neurodegenerative diseases, such as PD or ALS [77,78,79,80].

In light of this knowledge, the results we found for PM_2.5_ are not surprising: it can be hypothesized that these particles ignite a chain reaction involving oxidative stress, synaptic dysfunction, altered proteostasis and deposition of Aβ and phosphorylated tau protein, which are in turn the pathological hallmarks of AD [56,81]. It is thought that PM_2.5_ can exert its detrimental effect on brain health both by an indirect mechanism, due to its inflammatory action on the respiratory epithelium [82], and by a direct one, since it can reach the central nervous system through the systemic circulation or through the olfactory bulb [81,83]. For PM_10_, the higher diameter and the consequent lower penetration into airways of these particles could explain the lack of association we observed, if one admits that it was not a problem of lower sample size. Even if we did not find significant results for ozone and NO_2_, it is worth noting that the same pathophysiological mechanisms described for PM_2.5_ (oxidative stress, microglial activation, inflammation and astrocyte dysfunction) have been proposed for these gases too [84,85,86].

Our results are relevant, since the increase in AD risk we observed for a 10 μg/m^3^ increment of PM_2.5_ concentration should prompt relevant reflections in terms of public health policies, leading to a revision of current limits established by regulatory agencies. From this point of view, the low threshold (5 μg/m^3^) established by the guidelines of the World Health Organization (WHO) [87] seems to be more appropriate than the ones proposed by the US EPA (9 μg/m^3^) [88] and especially by the European Union (EU, currently 25 μg/m^3^) [89]. Moreover, it should be observed that the current limits for PM_2.5_ air concentration are not always fulfilled: for example, it is estimated that in 2023 and 2024 PM_2.5_ concentrations above the EU annual limit value were seen in multiple European regions (Italy, Turkey, and west Balkans) [89]. From this point of view, transitioning to a renewable source of energy, with subsequent avoidance of the use of fossil fuels, is a key step. It should also be noted that apart from reduction in emissions, the goal of lowering the exposure to PM_2.5_ and other pollutants can be reached also through strategies of air filtering, both outdoor (expanding green areas in polluted cities) and indoor (through appropriate heating, ventilation, and air conditioning systems).

We have to admit that the interpretation of our results needs caution, since we are aware of the limits of our review. Concerning the outcome, the main problem is the definition of AD. Even if the current approach in AD diagnosis is based on a purely biological (NIA-AA criteria) or clinical–biological basis (IWG criteria), in most of included articles neither of these sets of criteria were used; rather, a purely clinical or clinical–radiological approach was adopted. We recognize that this issue is a major limit of our review, since it could undermine the generalizability of our results and it could be linked to a high risk of selection bias. With regard to exposure, it is not clear if ground- and satellite-based data offer comparable results; moreover, length of exposure, which could potentially have a relevant impact on the risk increase, was not usually considered in included studies. In addition, the effect of co-exposure to multiple pollutants was not taken into account. Apart from this, it should be considered that the exposure measured in each geographical area does not necessarily match the individual exposure of people living in that area and thus associations can be biased; this is even more relevant because no data on indoor levels of pollutants were available. Another limit is the methodological heterogeneity of the included studies, in terms of population characteristics and definition of exposure and outcomes, which is probably the cause of the high heterogeneity we found in our meta-analyses, with I^2^ values ranging from 90 to 100%. As shown by the funnel plots and by the results of Egger’s test, publication bias was found in most of the analyses, showing that studies with smaller samples sizes have systematically different results than larger ones; this should be considered another caveat to the interpretation of our results. The presence of multiple studies on the same database is another issue, since it could inflate the impact of a dataset on the overall estimates; however, we tried to manage it through sensitivity analyses, which confirmed the results of the main analyses, thus limiting the concerns.

Our review shows important gaps in existing studies and points out some possible research goals for the future. The main one is the impact that the adoption of the current biological definitions of AD could have onto the association we have observed; we recognize that conducting such large-scale studies with the adoption of these diagnostic criteria, which imply the analysis of the cerebrospinal fluid and/or the use of nuclear and magnetic resonance imaging, is challenging and will probably request long time to get enough data, but it is necessary given the current diagnostic frame. Another issue to be explored in future studies is the interaction between air pollution and genetic predisposition to AD, since existing studies generally do not include apoE carrier status as a covariate for the calculation of adjusted HRs. The role of co-existing pathologies, both as parallel consequences of air pollution or as modulators of the impact of air pollution on AD pathology, is another theme that is not addressed in the current literature and that should be considered in future research. Moreover, apart from the impact of air pollution on the overall risk of developing AD, it would be interesting to investigate the influence on the age of AD onset and in the conversion rate from mild cognitive impairment to overt dementia.

## 5. Conclusions

We found that exposure to fine particulate matter significantly increases the risk of AD, at least when this disease is defined on clinical grounds; significant heterogeneity in included studies has to be taken into account in the interpretation of this result. Such a correlation was not observed for other pollutants, but given the existing pathophysiological evidence and the limits of our review, their role cannot be surely ruled out without further research. These findings underscore the importance of environmental policies aimed at reducing air pollution not only to prevent cardiovascular and respiratory diseases, but also to help mitigate cognitive decline, dementia, and other related health conditions.

## Figures and Tables

**Table 1:** Characteristics of included studies.

Study	Country	Study Design	Participants, *n*	Age ^a^	Female, *n* (%)	Data Source	Pollutant(s)
Qin 2025 [11]	United States	Retrospective cohort	50,053,399	65–95	27,779,626 (55.5)	For AD data: Medicare For pollution data: multiple sources	O_3_ PM_2.5_ NO_2_
Zheng 2025 [20]	United Kingdom	Cohort	217,336	64.1	114,521 (52.7)	For AD data: United Kingdom Biobank For pollution data: European Monitoring and Evaluation Program model for the UK	PM_2.5_
Zhu 2025 [21]	United States	Cohort	40,019,467	75	22,347,933 (55.84)	For AD data: Medicare and Medicaid For pollution data: multiple sources	PM_2.5_
Tian 2024 [22]	United Kingdom	Cohort	148,756	40–69	71,613 (48.14)	For AD data: United Kingdom Biobank For pollution data: European Study of Cohorts for Air Pollution Effects	PM_10_ PM_2.5_ NO_2_
Zhang 2024 [23]	United Kingdom	Cohort	155,828		77,649 (49.82)	For AD data: United Kingdom Biobank For pollution data: European Study of Cohorts for Air Pollution Effects	PM_2.5_ PM_10_ NO_2_
Ma 2023 [10]	China	Cohort	31,573	62.5	12,975 (41.11)	For AD data: Chinese Longitudinal Healthy Longevity Survey; Chinese Alzheimer’s Biomarker and Lifestyle study For pollution data: satellite-based observations	PM_2.5_
Yuan 2023 [24]	United Kingdom	Cohort	437,932	58–65	237,436 (54.21)	For AD data: United Kingdom Biobank For pollution data: Small Area Health Statistics Unit pollution	PM_2.5_ PM_10_ NO_x_
Zhu 2023 [25]	China	Cohort	29,025	63.3	17,180 (59.19)	For AD data: Yinzhou Health Information System For pollution data: China’s National Environmental Monitoring Center pollution	PM_10_ PM_2.5_ NO_2_
Parra 2022 [26]	United Kingdom	Cohort	187,194	64.1	98,459 (52.59)	For AD data: United Kingdom Biobank For pollution data: Small Area Health Statistics Unit	PM_2.5_
Shi 2023 [27]	United States	Cohort	37,719,448	65–75	22,405,352 (59.4)	For AD data: Medicare and Medicaid For pollution data: multiple sources	PM_2.5_
Yang 2022 [28]	China	Cohort	1545	68.2	806 (52.16)	For AD data: ad hoc questionnaires For pollution data: satellite-based observations	PM_2.5_
Zhang 2023 [2]	United Kingdom	Cohort	227,840	60.1	119,398 (52.4)	For AD data: United Kingdom Biobank data FOr pollution data: European Study of Cohorts for Air Pollution Effects data	PM_2.5_ PM_10_ NO_2_
Mortamais 2021 [29]	France	Cohort	7066	73.4	4359 (61.7)	For AD data: Three-City Study For pollution data: European Environment Agency AirBase network	PM_2.5_ NO_2_
Ran 2021 [30]	China	Cohort	59,349	65–85	38,914 (65.6)	For AD data: Chinese Elderly Health Service For pollution data: National Aeronautics and Space Administration	PM_2.5_
Rhew 2021 [31]	United States	Retrospective cohort	2,022,647	>65	1,177,393 (58.21)	For AD data: Healthcare Cost and Utilization Project’s State Inpatient Database For pollution data: National Aeronautics and Space Administration	PM_2.5_
Shi 2021 [32]	United States	Cohort	12,233,371	65–114	7,205,455 (58.9)	For AD data: Medicare For pollution data: multiple sources	PM_2.5_ PM_10_ NO_2_
Shi 2020 [33]	United States	Cohort	63,038,019	69–90	34,742,032 (55.11)	For AD data: Medicare US Environmental Protection Agency pollution data For pollution data: IMPROVE monitoring network	PM_2.5_
Smargiassi 2020 [34]	Canada	Cohort	1,807,133	65–85	995,495 (55.09)	For AD data: Québec Integrated Chronic Disease Surveillance System For pollution data: Ground- and satellite-based observations	PM_2.5_ NO_2_
Yuchi 2020 [35]	Canada	Cohort	678,800	45–84	-	For AD data: PharmaNet network For pollution data: CanMap road network	PM_2.5_ NO_2_
Cerza 2019 [36]	Italy	Cohort	350,844	74.5	204,900 (58.40)	For AD data: Rome Longitudinal Study; hospital discharge registry For pollution data: European Study of Cohorts for Air Pollution Effects	PM_2.5_ PM_10_ NO_2_ O_3_
Carey 2018 [37]	United Kingdom	Cohort	130,978	50–79	65,848 (50.27)	For AD data: Clinical Practice Research Datalink For pollution data: London Atmospheric Emissions Inventory	PM_2.5_ NO_2_ O_3_
Oudin 2018 [38]	Sweden	Cohort	1806	55–85	1033 (57.20)	For AD data: Betula study For pollution data: Statistics Sweden	PM_2.5_
Culqui 2017 [39]	Spain	Cohort	754,005	≥60	-	For AD data: Madrid Hospital Morbidity Survey For pollution data: Madrid Municipal Air Quality Monitoring Grid	PM_2.5_
Kioumourtzoglou 2016 [40]	United States	Cohort	9,817,806	75.6	5,625,602 (57.29)	For AD data: Medicare For pollution data: US Environmental Protection Agency	PM_2.5_
Oudin 2016 [41]	Sweden	Cohort	1806	55–85	1033 (57.20)	For AD data: Betula study For pollution data: ad hoc ground-based observations	NO_2_
Jung 2015 [42]	Taiwan	Cohort	95,690	≥65	44,119 (46.10)	For AD data: Longitudinal Health Insurance Database 2000 For pollution data: Taiwan Environmental Protection Agency	O_3_ PM_2.5_ PM_10_
Wu 2015 [1]	Taiwan	Case–control	871	≥60	490 (56.26)	For AD data: hospital records For pollution data: Taiwan Environmental Protection Agency	O_3_ PM_10_

^a^ mean or range; AD: Alzheimer’s disease.

**Table 2:** Studies on the association between PM_2.5_ and Alzheimer’s disease.

Study	Increase in Pollutant Concentration	Effect Size Type	Crude Effect Size (CI)	Adjusted Effect Size (CI)	Covariates for Adjustment
Qin 2025 [6]	4.16 μg/m^3^	HR	1.15 (1.14–1.16)	-	-
Zheng 2025 [20]	1.9 μg/m^3^	HR	-	1.12 (1.08–1.16)	Age, sex, education, BMI, smoking, diabetes, hypertension
Zhu 2025 [21]	1 μg/m^3^	HR	1.03 (1.03–1.045)	-	-
Tian 2024 [22]	1.26 μg/m^3^	HR	1.11 (1.07–1.16)	1.07 (1.02–1.12)	Age, sex, ethnicity, education, socioeconomic status, BMI, smoking
Zhang 2024 [23]	1 μg/m^3^	HR	1.1 (1.04–1.15)	-	-
Ma 2023 [10]	20 μg/m^3^	HR	1.07 (1–1.14)	1.12 (1.04–1.2)	Age, sex, educational level, comorbidities, lifestyles, and socioeconomic factors
Yuan 2023 [24]	10 μg/m^3^	HR	1.17 (1.02–1.35)	1.13 (0.97–1.31)	Ethnicity, sex, education, age, smoking
Zhu 2023 [25]	5.32 μg/m^3^	HR	-	1.41 (1.09–1.84)	Age, sex, occupation, educational level, smoking, alcohol, BMI
Parra 2022 [26]	1 μg/m^3^	HR	1.17 (1.06–1.29)	-	-
Shi 2023 [27]	1 μg/m^3^	HR	-	1.06 (1.099–1.114)	Socioeconomic status
Yang 2022 [28]	10 μg/m^3^	HR	-	1.02 (1.01–1.09)	Age, sex
Zhang 2023 [2]	1.3 μg/m^3^	HR	1.1(1.03–1.17)	1.03 (0.955–1.12)	Educational level, BMI, smoking, alcohol, physical activity, fruit and vegetable intake, family history of dementia
Mortamais 2021 [29]	5 μg/m^3^	HR	1.33 (1.14–1.56)	1.20 (1.09–1.32)	Sex, education, smoking, alcohol
Ran 2021 [30]	3.8 μg/m^3^	HR	1.09 (1–1.18)	1.03 (0.94–1.12)	Age, sex, BMI, smoking, alcohol
Rhew 2021 [31]	10.27 μg/m^3^	OR		1.35 (1.24–1.48)	Age, sex, ethnicity, socioeconomic status
Shi 2021 [32]	3.2 μg/m^3^	HR	1.078 (1.070–1.086)	1.078 (1.070–1.086)	Co-pollutants
Shi 2020 [33]	5 μg/m^3^	HR	1.13 (1.12–1.14)	-	-
Smargiassi 2020 [34]	3.9 μg/m^3^	HR	1.024 (1.017–1.031)	1.016 (1–1.03)	Sex
Yuchi 2020 [35]	1.54 μg/m^3^	OR	-	0.9 (0.76–1.07)	Age, sex, ethnicity, education
Cerza 2019 [36]	5 μg/m^3^	HR	-	0.91 (0.85–0.97)	Age, education, socioeconomic status
Carey 2018 [37]	0.9 μg/m^3^	HR	-	1.1 (1.02–1.18)	Age, sex, smoking, BMI, alcohol
Oudin 2018 [38]	1 μg/m^3^	HR	1.05 (0.7–1.57)	1.55 (1–2.41)	Physical activity, smoking, sex, BMI, age
Culqui 2017 [39]	20 μg/m^3^	RR	1.38 (1.15–1.65)		
Kioumourtzoglou 2016 [40]	1 μg/m^3^	HR	1.15 (1.11–1.19)	-	-
Jung 2015 [42]	4.34 μg/m^3^	HR	2.41 (2.24–2.59)	2.38 (2.21–2.56)	Age, gender, socioeconomic state, pathologies

HR: hazard ratio; OR: odds ratio; RR: relative risk; BMI: body mass index.

**Table 3:** Studies on the association between PM_10_ and Alzheimer’s disease.

Study	Increase in Pollutant Concentration	Effect Size Type	Crude Effect Size (CI)	Adjusted Effect Size (CI)	Covariates for Adjustment
Tian 2024 [22]	2.3 μg/m^3^	HR	1.14 (1.09–1.19)	1.10 (1.04–1.15)	Age, sex, ethnicity, education, socioeconomic status, BMI, smoking
Zhang 2024 [23]	1 μg/m^3^	HR	1.03 (0.98–1.09)	-	-
Yuan 2023 [24]	15 μg/m^3^	HR	1.12 (0.94–1.34)	1.06 (0.88–1.27)	Age, sex, ethnicity, education, socioeconomic status, alcohol
Zhu 2023 [25]	1 μg/m^3^	HR	-	1.16 (0.99–1.36)	Age, sex, occupation, educational level, smoking, alcohol, BMI
Zhang 2023 [2]	2.3 μg/m^3^	HR	1.06 (0.98–1.14)	1.07 (0.98–1.17)	Educational level, BMI, smoking, alcohol, physical activity, fruit and vegetable intake, family history of dementia
Cerza 2019 [36]	10 μg/m^3^	HR	0.95 (0.91–0.99)	-	-
Wu 2015 [1]	49.23 μg/m^3^	OR	-	4.17 (2.31–7.54)	Age, education, BMI

HR: hazard ratio; OR: odds ratio; BMI: body mass index.

**Table 4:** Studies on the association between NO_2_ and Alzheimer’s disease.

Study	Increase in Pollutant Concentration	Effect Size Type	Crude Effect Size (CI)	Adjusted Effect Size (CI)	Covariates for Adjustment
Qin 2025 [11]	12.01 μg/m^3^	HR	1.145 (1.13–1.15)	-	-
Tian 2024 [22]	10.75 μg/m^3^	HR	1.14 (1.09–1.19)	1.08 (1.03–1.14)	Age, sex, ethnicity, education, socioeconomic status, BMI, smoking
Zhang 2024 [23]	1 μg/m^3^	HR	1.09 (1.03–1.14)	-	-
Zhu 2023 [25]	11.09 μg/m^3^	HR	-	1.09 (0.73–1.61)	Age, sex, occupation, educational level, smoking, alcohol, BMI
Parra 2022 [26]	1 μg/m^3^	HR	1.15 (1.04–1.28)	-	-
Zhang 2023 [2]	10.5 μg/m^3^	HR	1.15 (1.08–1.23)	1.16 (1.06–1.26)	Educational level, BMI, smoking, alcohol, physical activity, fruit and vegetable intake, family history of dementia
Mortamais 2021 [29]	5 μg/m^3^	HR	0.94 (0.87–1.01)	1.01 (0.96–1.05)	Sex, education, smoking, alcohol
Shi 2021 [32]	11.6 μg/m^3^	HR	1.05 (1.04–1.05)	1.03 (1.02–1.04)	Co-pollutants
Smargiassi 2020 [34]	13.26 ppb	HR	1.023 (1.01–1.03)	1005 (0.99–1.01)	Sex
Cerza 2019 [36]	10 μg/m^3^	HR	0.91 (0.89–0.94)	-	-
Carey 2018 [37]	7.5 μg/m^3^	HR	-	1.23 (1.07–1.43)	Age, sex, smoking, BMI, alcohol
Oudin 2016 [41]	10 μg/m^3^	HR	-	1.05 (0.97–1.15)	Age, education, physical activity, smoking, sex, BMI, alcohol

ppb: parts per billion; HR: hazard ratio; BMI: body mass index.

**Table 5:** Studies on the association between O_3_ and Alzheimer disease.

Study	Increase in Pollutant Concentration	Effect Size Type	Crude Effect Size (CI)	Adjusted Effect Size (CI)	Covariates for Adjustment
Qin 2025 [11]	9.8 ppb	HR	1.057 (1.05–1.06)	-	-
Shi 2021 [32]	5.3 ppb	HR	0.99 (0.99–1)	0.98 (0.97–0.98)	Co-pollutants
Cerza 2019 [36]	10 μg/m^3^	HR	0.98 (0.95–1.02)	-	-
Carey 2018 [37]	5.6 μg/m^3^	HR	-	0.78 (0.66–0.92)	Age, sex, smoking, BMI, alcohol
Jung 2015 [42]	10.91 ppb	HR	3.11 (2.31–3.32)	3.12 (2.92–3.33)	Age, gender, socioeconomic state, pathologies
Wu 2015 [1]	21.56 μg/m^3^	OR	-	2 (1.14–3.5)	Age, education, BMI

ppb: parts per billion; HR: hazard ratio; OR: odds ratio; BMI: body mass index.

## Data Availability

Not applicable.

## Supplementary Materials

The following supporting information can be downloaded at https://www.mdpi.com/article/10.3390/jcm15114163/s1, Table S1: PRISMA checklist; Table S2: PubMed/MEDLINE search history; Table S3: Embase (via OVID) search history; Table S4: Scopus search history; Table S5: Characteristics of excluded studies; Table S6: Quality appraisal for included articles; Figure S1: Funnel plot for the studies on the association between PM_2.5_ and AD risk; Figure S2: Funnel plot for the studies on the association between PM_10_ and AD risk; Figure S3: Funnel plot for the studies on the association between NO_2_ and AD risk; Figure S4: Funnel plot for the studies on the association between O_3_ and AD risk; Figure S5: Sensitivity analysis: forest plot for the association between PM_2.5_ and AD risk; Figure S6: Sensitivity analysis: forest plot for the association between PM_10_ and AD risk; Figure S7: Sensitivity analysis: forest plot for the association between NO_2_ and AD risk; Figure S8: Sensitivity analysis: forest plot for the association between O_3_ and AD risk.

## References

[1] Wu Y., Lin Y., Yu H., Chen J., Chen T., Sun Y., Wen L., Yip P., Chu Y., Chen Y. (2015). Association between Air Pollutants and Dementia Risk in the Elderly. Alzheimers Dement. Diagn. Assess. Dis. Monit..

[2] Zhang Z., Chen L., Wang X., Wang C., Yang Y., Li H., Cai M., Lin H. (2023). Associations of Air Pollution and Genetic Risk with Incident Dementia: A Prospective Cohort Study. Am. J. Epidemiol..

[3] Wimo A., Seeher K., Cataldi R., Cyhlarova E., Dielemann J.L., Frisell O., Guerchet M., Jönsson L., Malaha A.K., Nichols E. (2023). The Worldwide Costs of Dementia in 2019. Alzheimer’s Dement..

[4] Breijyeh Z., Karaman R. (2020). Comprehensive Review on Alzheimer’s Disease: Causes and Treatment. Molecules.

[5] McKhann G., Drachman D., Folstein M., Katzman R., Price D., Stadlan E.M. (1984). Clinical Diagnosis of Alzheimer’s Disease: Report of the NINCDS-ADRDA Work Group under the Auspices of Department of Health and Human Services Task Force on Alzheimer’s Disease. Neurology.

[6] Jack C.R., Andrews J.S., Beach T.G., Buracchio T., Dunn B., Graf A., Hansson O., Ho C., Jagust W., McDade E. (2024). Revised Criteria for Diagnosis and Staging of Alzheimer’s Disease: Alzheimer’s Association Workgroup. Alzheimer’s Dement..

[7] Dubois B., Villain N., Schneider L., Fox N., Campbell N., Galasko D., Kivipelto M., Jessen F., Hanseeuw B., Boada M. (2024). Alzheimer Disease Is a Clinical-Biological Construct: An IWG Recommendation. JAMA Neurol..

[8] Wang T., Guo Z., Du Y., Xiong M., Yang Z., Ren L., He L., Jiang Y., McClure M.A., Mu Q. (2021). Effects of Noninvasive Brain Stimulation (NIBS) on Cognitive Impairment in Mild Cognitive Impairment and Alzheimer Disease: A Meta-Analysis. Alzheimer Dis. Assoc. Disord..

[9] Van Dyck C.H., Swanson C.J., Aisen P., Bateman R.J., Chen C., Gee M., Kanekiyo M., Li D., Reyderman L., Cohen S. (2023). Lecanemab in Early Alzheimer’s Disease. N. Engl. J. Med..

[10] Ma Y.-H., Chen H.-S., Liu C., Feng Q.-S., Feng L., Zhang Y.-R., Hu H., Dong Q., Tan L., Kan H.-D. (2023). Association of Long-Term Exposure to Ambient Air Pollution with Cognitive Decline and Alzheimer’s Disease–Related Amyloidosis. Biol. Psychiatry.

[11] Qin M.M., Khoshnevis N., Dominici F., Braun D., Zanobetti A., Mork D. (2025). Comparing Traditional and Causal Inference Methodologies for Evaluating Impacts of Long-Term Air Pollution Exposure on Hospitalization with Alzheimer Disease and Related Dementias. Am. J. Epidemiol..

[12] Best Rogowski C.B., Bredell C., Shi Y., Tien-Smith A., Szybka M., Fung K.W., Hong L., Phillips V., Jovanovic Andersen Z., Sharp S.J. (2025). Long-Term Air Pollution Exposure and Incident Dementia: A Systematic Review and Meta-Analysis. Lancet Planet. Health.

[13] Moher D., Liberati A., Tetzlaff J., Altman D.G., The PRISMA Group (2009). Preferred Reporting Items for Systematic Reviews and Meta-Analyses: The PRISMA Statement. Ann. Intern. Med..

[14] Haddaway N.R., Page M.J., Pritchard C.C., McGuinness L.A. (2022). PRISMA2020: An R Package and Shiny App for Producing PRISMA 2020-compliant Flow Diagrams, with Interactivity for Optimised Digital Transparency and Open Synthesis. Campbell Syst. Rev..

[15] Clark J.M., Sanders S., Carter M., Honeyman D., Cleo G., Auld Y., Booth D., Condron P., Dalais C., Bateup S. (2020). Improving the Translation of Search Strategies Using the Polyglot Search Translator: A Randomized Controlled Trial. J. Med. Libr. Assoc..

[16] Clark J., Glasziou P., Del Mar C., Bannach-Brown A., Stehlik P., Scott A.M. (2020). A Full Systematic Review Was Completed in 2 Weeks Using Automation Tools: A Case Study. J. Clin. Epidemiol..

[17] Forbes C., Greenwood H., Carter M., Clark J. (2024). Automation of Duplicate Record Detection for Systematic Reviews: Deduplicator. Syst. Rev..

[18] Ouzzani M., Hammady H., Fedorowicz Z., Elmagarmid A. (2016). Rayyan—A Web and Mobile App for Systematic Reviews. Syst. Rev..

[19] Fu P., Yung K.K.L. (2020). Air Pollution and Alzheimer’s Disease: A Systematic Review and Meta-Analysis. J. Alzheimer’s Dis..

[20] Zheng L., Su B., Cui F.-P., Li D., Ma Y., Xing M., Tang L., Wang J., Tian Y., Zheng X. (2025). Long-Term Exposure to PM_2.5_ Constituents, Genetic Susceptibility, and Incident Dementia: A Prospective Cohort Study among 0.2 Million Older Adults. Environ. Sci. Technol..

[21] Zhu Q., Deng Y.-L., Liu Y., Steenland K. (2025). Associations between Ultrafine Particles and Incident Dementia in Older Adults. Environ. Sci. Technol..

[22] Tian F., Qian Z., Zhang Z., Liu Y., Wu G., Wang C., McMillin S.E., Bingheim E., Lin H. (2024). Air Pollution, APOE Genotype and Risk of Dementia among Individuals with Cardiovascular Diseases: A Population-Based Longitudinal Study. Environ. Pollut..

[23] Zhang Y., Fu Y., Guan X., Wang C., Fu M., Xiao Y., Hong S., Zhou Y., Liu C., Zhong G. (2024). Associations of Ambient Air Pollution Exposure and Lifestyle Factors with Incident Dementia in the Elderly: A Prospective Study in the UK Biobank. Environ. Int..

[24] Yuan S., Huang X., Zhang L., Ling Y., Tan S., Peng M., Xu A., Lyu J. (2023). Associations of Air Pollution with All-Cause Dementia, Alzheimer’s Disease, and Vascular Dementia: A Prospective Cohort Study Based on 437,932 Participants from the UK Biobank. Front. Neurosci..

[25] Zhu Z., Yang Z., Yu L., Xu L., Wu Y., Zhang X., Shen P., Lin H., Shui L., Tang M. (2023). Residential Greenness, Air Pollution and Incident Neurodegenerative Disease: A Cohort Study in China. Sci. Total Environ..

[26] Parra K.L., Alexander G.E., Raichlen D.A., Klimentidis Y.C., Furlong M.A. (2022). Exposure to Air Pollution and Risk of Incident Dementia in the UK Biobank. Environ. Res..

[27] Shi L., Zhu Q., Wang Y., Hao H., Zhang H., Schwartz J., Amini H., Van Donkelaar A., Martin R.V., Steenland K. (2023). Incident Dementia and Long-Term Exposure to Constituents of Fine Particle Air Pollution: A National Cohort Study in the United States. Proc. Natl. Acad. Sci. USA.

[28] Yang L., Wan W., Yu C., Xuan C., Zheng P., Yan J. (2022). Associations between PM_2.5_ Exposure and Alzheimer’s Disease Prevalence Among Elderly in Eastern China. Environ. Health.

[29] Mortamais M., Gutierrez L.-A., De Hoogh K., Chen J., Vienneau D., Carrière I., Letellier N., Helmer C., Gabelle A., Mura T. (2021). Long-Term Exposure to Ambient Air Pollution and Risk of Dementia: Results of the Prospective Three-City Study. Environ. Int..

[30] Ran J., Schooling C.M., Han L., Sun S., Zhao S., Zhang X., Chan K.-P., Guo F., Lee R.S., Qiu Y. (2021). Long-Term Exposure to Fine Particulate Matter and Dementia Incidence: A Cohort Study in Hong Kong. Environ. Pollut..

[31] Rhew S.H., Kravchenko J., Lyerly H.K. (2021). Exposure to Low-Dose Ambient Fine Particulate Matter PM_2.5_ and Alzheimer’s Disease, Non-Alzheimer’s Dementia, and Parkinson’s Disease in North Carolina. PLoS ONE.

[32] Shi L., Steenland K., Li H., Liu P., Zhang Y., Lyles R.H., Requia W.J., Ilango S.D., Chang H.H., Wingo T. (2021). A National Cohort Study (2000–2018) of Long-Term Air Pollution Exposure and Incident Dementia in Older Adults in the United States. Nat. Commun..

[33] Shi L., Wu X., Danesh Yazdi M., Braun D., Abu Awad Y., Wei Y., Liu P., Di Q., Wang Y., Schwartz J. (2020). Long-Term Effects of PM2·5 on Neurological Disorders in the American Medicare Population: A Longitudinal Cohort Study. Lancet Planet. Health.

[34] Smargiassi A., Sidi E.A.L., Robert L.-E., Plante C., Haddad M., Gamache P., Burnett R., Goudreau S., Liu L., Fournier M. (2020). Exposure to Ambient Air Pollutants and the Onset of Dementia in Québec, Canada. Environ. Res..

[35] Yuchi W., Sbihi H., Davies H., Tamburic L., Brauer M. (2020). Road Proximity, Air Pollution, Noise, Green Space and Neurologic Disease Incidence: A Population-Based Cohort Study. Environ. Health.

[36] Cerza F., Renzi M., Gariazzo C., Davoli M., Michelozzi P., Forastiere F., Cesaroni G. (2019). Long-Term Exposure to Air Pollution and Hospitalization for Dementia in the Rome Longitudinal Study. Environ. Health.

[37] Carey I.M., Anderson H.R., Atkinson R.W., Beevers S.D., Cook D.G., Strachan D.P., Dajnak D., Gulliver J., Kelly F.J. (2018). Are Noise and Air Pollution Related to the Incidence of Dementia? A Cohort Study in London, England. BMJ Open.

[38] Oudin A., Segersson D., Adolfsson R., Forsberg B. (2018). Association between Air Pollution from Residential Wood Burning and Dementia Incidence in a Longitudinal Study in Northern Sweden. PLoS ONE.

[39] Culqui D.R., Linares C., Ortiz C., Carmona R., Díaz J. (2017). Association between Environmental Factors and Emergency Hospital Admissions Due to Alzheimer’s Disease in Madrid. Sci. Total Environ..

[40] Kioumourtzoglou M.-A., Schwartz J.D., Weisskopf M.G., Melly S.J., Wang Y., Dominici F., Zanobetti A. (2016). Long-Term PM_2.5_ Exposure and Neurological HospitalAdmissions in the Northeastern United States. Environ. Health Perspect..

[41] Oudin A., Forsberg B., Adolfsson A.N., Lind N., Modig L., Nordin M., Nordin S., Adolfsson R., Nilsson L.-G. (2016). Traffic-Related Air Pollution and Dementia Incidence in Northern Sweden: A Longitudinal Study. Environ. Health Perspect..

[42] Jung C.-R., Lin Y.-T., Hwang B.-F. (2015). Ozone, Particulate Matter, and Newly Diagnosed Alzheimer’s Disease: A Population-Based Cohort Study in Taiwan. J. Alzheimer’s Dis..

[43] Guo C., Wu D., Yang J., Lu X., Chen X.Y., Ma J., Lin C., Lau A.K.H., Jin Y., Li R. (2025). Ambient Air Pollution and Alzheimer’s Disease and Other Dementias: A Global Study between 1990 and 2019. BMC Public Health.

[44] Thompson R., Tong X., Shen X., Ran J., Sun S., Yao X.I., Shen C. (2025). Longitudinal Associations between Air Pollution and Incident Dementia as Mediated by MRI-Measured Brain Volumes in the UK Biobank. Environ. Int..

[45] Casey E., Li Z., Liang D., Ebelt S., Levey A.I., Lah J.J., Wingo T.S., Hüls A. (2024). Association between Fine Particulate Matter Exposure and Cerebrospinal Fluid Biomarkers of Alzheimer’s Disease among a Cognitively Healthy Population-Based Cohort. Environ. Health Perspect..

[46] Zhang H., Wang Y., Li H., Zhu Q., Ma T., Liu Y., Steenland K. (2025). The Role of the Components of PM_2.5_ in the Incidence of Alzheimer’s Disease and Related Disorders. Environ. Int..

[47] Ji Q., Liu Q., Xu Y., Xu M., Zhan Y. (2026). Long-Term Exposure to Residential Greenspace, Bluespace, Traffic, and Air Pollutants with All-Cause and Cause-Specific Dementia: A Prospective Cohort Study. Ecotoxicol. Environ. Saf..

[48] Blanco M.N., Shaffer R.M., Li G., Adar S.D., Carone M., Szpiro A.A., Kaufman J.D., Larson T.V., Hajat A., Larson E.B. (2024). Traffic-Related Air Pollution and Dementia Incidence in the Adult Changes in Thought Study. Environ. Int..

[49] Ge R., Wang Y., Zhang Z., Sun H., Chang J. (2024). Association of Long-Term Exposure to Various Ambient Air Pollutants, Lifestyle, and Genetic Predisposition with Incident Cognitive Impairment and Dementia. BMC Public Health.

[50] Gialluisi A., Costanzo S., Veronesi G., Cembalo A., Tirozzi A., Falciglia S., Ricci M., Martone F., Zazzaro G., Ferrario M.M. (2023). Prominent Role of PM_10_ but Not of Circulating Inflammation in the Link between Air Pollution and the Risk of Neurodegenerative Disorders. medRxiv.

[51] Semmens E.O., Leary C.S., Fitzpatrick A.L., Ilango S.D., Park C., Adam C.E., DeKosky S.T., Lopez O., Hajat A., Kaufman J.D. (2023). Air Pollution and Dementia in Older Adults in the Ginkgo Evaluation of Memory Study. Alzheimer’s Dement..

[52] De Crom T.O.E., Ginos B.N.R., Oudin A., Ikram M.K., Voortman T., Ikram M.A. (2023). Air Pollution and the Risk of Dementia: The Rotterdam Study. J. Alzheimer’s Dis..

[53] Andersson J., Sundström A., Nordin M., Segersson D., Forsberg B., Adolfsson R., Oudin A. (2023). PM_2.5_ and Dementia in a Low Exposure Setting: The Influence of Odor Identification Ability and APOE. J. Alzheimer’s Dis..

[54] Petkus A.J., Salminen L.E., Wang X., Driscoll I., Millstein J., Beavers D.P., Espeland M.A., Braskie M.N., Thompson P.M., Casanova R. (2023). Alzheimer’s Related Neurodegeneration Mediates Air Pollution Effects on Medial Temporal Lobe Atrophy. medRxiv.

[55] Bishop K.C., Ketcham J.D., Kuminoff N.V. (2023). Hazed and Confused: The Effect of Air Pollution on Dementia. Rev. Econ. Stud..

[56] Lee Y., Yoon S., Yoon S.H., Kang S.W., Jeon S., Kim M., Shin D.A., Nam C.M., Ye B.S. (2023). Air Pollution Is Associated with Faster Cognitive Decline in Alzheimer’s Disease. Ann. Clin. Transl. Neurol..

[57] Rodriguez-Loureiro L., Gadeyne S., Bauwelinck M., Lefebvre W., Vanpoucke C., Casas L. (2022). Long-Term Exposure to Residential Greenness and Neurodegenerative Disease Mortality among Older Adults: A 13-Year Follow-up Cohort Study. Environ. Health.

[58] Wang X., Younan D., Millstein J., Petkus A.J., Garcia E., Beavers D.P., Espeland M.A., Chui H.C., Resnick S.M., Gatz M. (2022). Association of Improved Air Quality with Lower Dementia Risk in Older Women. Proc. Natl. Acad. Sci. USA.

[59] Åström D.O., Adolfsson R., Segersson D., Forsberg B., Oudin A. (2021). Local Contrasts in Concentration of Ambient Particulate Air Pollution (PM_2.5_) and Incidence of Alzheimer’s Disease and Dementia: Results from the Betula Cohort in Northern Sweden. J. Alzheimer’s Dis..

[60] Alemany S., Crous-Bou M., Vilor-Tejedor N., Milà-Alomà M., Suárez-Calvet M., Salvadó G., Cirach M., Arenaza-Urquijo E.M., Sanchez-Benavides G., Grau-Rivera O. (2021). Associations between Air Pollution and Biomarkers of Alzheimer’s Disease in Cognitively Unimpaired Individuals. Environ. Int..

[61] Sullivan K.J., Ran X., Wu F., Chang C.-C.H., Sharma R., Jacobsen E., Berman S., Snitz B.E., Sekikawa A., Talbott E.O. (2021). Ambient Fine Particulate Matter Exposure and Incident Mild Cognitive Impairment and Dementia. J. Am. Geriatr. Soc..

[62] Younan D., Wang X., Casanova R., Barnard R., Gaussoin S.A., Saldana S., Petkus A.J., Beavers D.P., Resnick S.M., Manson J.E. (2021). PM_2.5_ Associated with Gray Matter Atrophy Reflecting Increased Alzheimer Risk in Older Women. Neurology.

[63] Crous-Bou M., Gascon M., Gispert J.D., Cirach M., Sánchez-Benavides G., Falcon C., Arenaza-Urquijo E.M., Gotsens X., Fauria K., Sunyer J. (2020). Impact of Urban Environmental Exposures on Cognitive Performance and Brain Structure of Healthy Individuals at Risk for Alzheimer’s Dementia. Environ. Int..

[64] Grande G., Ljungman P.L.S., Eneroth K., Bellander T., Rizzuto D. (2020). Association Between Cardiovascular Disease and Long-Term Exposure to Air Pollution with the Risk of Dementia. JAMA Neurol..

[65] Lee J.H., Byun M.S., Yi D., Ko K., Jeon S.Y., Sohn B.K., Lee J.-Y., Lee Y., Joung H., for the KBASE Research Group (2020). Long-Term Exposure to PM_10_ and in Vivo Alzheimer’s Disease Pathologies. J. Alzheimer’s Dis..

[66] Paul K.C., Haan M., Yu Y., Inoue K., Mayeda E.R., Dang K., Wu J., Jerrett M., Ritz B. (2020). Traffic-Related Air Pollution and Incident Dementia: Direct and Indirect Pathways Through Metabolic Dysfunction. J. Alzheimer’s Dis. JAD.

[67] Younan D., Petkus A.J., Widaman K.F., Wang X., Casanova R., Espeland M.A., Gatz M., Henderson V.W., Manson J.E., Rapp S.R. (2020). Particulate Matter and Episodic Memory Decline Mediated by Early Neuroanatomic Biomarkers of Alzheimer’s Disease. Brain.

[68] Li R.-L., Ho Y.-C., Luo C.-W., Lee S.-S., Kuan Y.-H. (2019). Influence of PM_2.5_ Exposure Level on the Association between Alzheimer’s Disease and Allergic Rhinitis: A National Population-Based Cohort Study. Int. J. Environ. Res. Public Health.

[69] Chen H., Kwong J.C., Copes R., Hystad P., van Donkelaar A., Tu K., Brook J.R., Goldberg M.S., Martin R.V., Murray B.J. (2017). Exposure to Ambient Air Pollution and the Incidence of Dementia: A Population-Based Cohort Study. Environ. Int..

[70] Wilker E.H., Osman M., Weisskopf M.G. (2023). Ambient Air Pollution and Clinical Dementia: Systematic Review and Meta-Analysis. BMJ.

[71] Heneka M.T., van der Flier W.M., Jessen F., Hoozemanns J., Thal D.R., Boche D., Brosseron F., Teunissen C., Zetterberg H., Jacobs A.H. (2025). Neuroinflammation in Alzheimer Disease. Nat. Rev. Immunol..

[72] Long C., Fritts A., Broadway J., Brawman-Mintzer O., Mintzer J. (2025). Neuroinflammation: A Driving Force in the Onset and Progression of Alzheimer’s Disease. J. Clin. Med..

[73] Zhang W., Xiao D., Mao Q., Xia H. (2023). Role of Neuroinflammation in Neurodegeneration Development. Signal Transduct. Target. Ther..

[74] Kostrzewska P., Kuca P., Witek P., Małyszko J., Madetko Alster N., Alster P. (2025). SGLT-2 Inhibitors in the Prevention and Progression of Neurodegenerative Diseases: A Narrative Review. Neurol. Ther..

[75] Al-kuraishy H.M., Jabir M.S., Albuhadily A.K., Al-Gareeb A.I., Rafeeq M.F. (2023). The Link between Metabolic Syndrome and Alzheimer Disease: A Mutual Relationship and Long Rigorous Investigation. Ageing Res. Rev..

[76] Yang F., Yang H., Han S., Wang J., Gao W., Ye Q., Zhang M., Yang Y., Li H. (2026). Microglial Metabolic Reprogramming in Alzheimer’s Disease: Pathways, Mechanisms, and Therapeutic Implications. Ageing Res. Rev..

[77] Antonioni A., Govoni V., Brancaleoni L., Donà A., Granieri E., Bergamini M., Gerdol R., Pugliatti M. (2023). Amyotrophic Lateral Sclerosis and Air Pollutants in the Province of Ferrara, Northern Italy: An Ecological Study. Int. J. Environ. Res. Public Health.

[78] Krzyzanowski B., Mullan A.F., Turcano P., Camerucci E., Bower J.H., Savica R. (2024). Air Pollution and Parkinson Disease in a Population-Based Study. JAMA Netw. Open.

[79] Roy R., D’Angiulli A. (2024). Air Pollution and Neurological Diseases, Current State Highlights. Front. Neurosci..

[80] Zhang B., Weuve J., Langa K.M., D’Souza J., Szpiro A., Faul J., Mendes de Leon C., Gao J., Kaufman J.D., Sheppard L. (2023). Comparison of Particulate Air Pollution from Different Emission Sources and Incident Dementia in the US. JAMA Intern. Med..

[81] Rubio C., López-Landa A., Serrano-García N., Romo-Parra H., Rubio-Osornio M. (2025). Impact of Particulate Matter 2.5 on Neurological Diseases: Insights into Pathophysiological and Molecular Mechanisms. J. Toxicol..

[82] Costa L.G., Cole T.B., Coburn J., Chang Y.-C., Dao K., Roqué P.J. (2017). Neurotoxicity of Traffic-Related Air Pollution. Neurotoxicology.

[83] Wei S., Xu T., Cao M., Wang H., Song Y., Yin D. (2024). The Constituent-Dependent Translocation Mechanism for PM_2.5_ to Travel through the Olfactory Pathway. Environ. Health.

[84] Ahmed C., Greve H.J., Garza-Lombo C., Malley J.A., Johnson J.A., Oblak A.L., Block M.L. (2024). Peripheral HMGB1 Is Linked to O3 Pathology of Disease-Associated Astrocytes and Amyloid. Alzheimer’s Dement. J. Alzheimer’s Assoc..

[85] Marin-Castañeda L.A., Gonzalez-Garibay G., Garcia-Quintana I., Pacheco-Aispuro G., Rubio C. (2024). Mechanisms of Ozone-Induced Neurotoxicity in the Development and Progression of Dementia: A Brief Review. Front. Aging Neurosci..

[86] Lane M., Oyster E., Luo Y., Wang H. (2025). The Effects of Air Pollution on Neurological Diseases: A Narrative Review on Causes and Mechanisms. Toxics.

[87] World Health Organization (2021). WHO Global Air Quality Guidelines.

[88] U.S. Environmental Protection Agency (2024). Reconsideration of the National Ambient Air Quality Standards for Particulate Matter.

[89] Targa J., Colina M., Banyuls L., Gonzalez Ortiz A., Soares J. (2025). ETC HE Report 2025/1: Status Report of Air Quality in Europe for Year 2024, Using Validated and Up-to-Date Data.

