# Supplementary material for: Air Pollution and Alzheimer’s Disease: A Systematic Review and Meta-Analysis
Source: J Clin Med. 2026 May 28;15(11):4163. doi: 10.3390/jcm15114163 (PMC13257598; doi:10.3390/jcm15114163)
Supplement: Supplementary file 1 [file jcm-15-04163-s001.zip › Table S2.pdf]

Table S2. PubMed/MEDLINE Search History

Search	PubMed/MEDLINE Query – 18th May, 2025	Items found
#14	#12 AND #13	320
#13	#4 OR #5 OR #6 OR #7 OR #8 OR #9 OR #10	1073171
#12	#1 AND #11	420
#11	#2 OR #3	152263
#10	"Smog"[Mesh] OR “Smog”[tiab]	2057
#9	"Vehicle Emissions"[Mesh] OR “Vehicle Emission*”[tiab] OR “Vehicular Emission*”[tiab] OR “Diesel Exhaust”[tiab] OR “Automobile Exhaust”[tiab] OR “Engine Exhaust”[tiab] OR “Traffic-Related Pollutant*”[tiab] OR “Transportation Emission*”[tiab]	15565
#8	"Carbon Monoxide"[Mesh] OR “Carbon Monoxide”[tiab] OR “CO”[tiab]	909209
#7	"Nitrogen Dioxide"[Mesh] OR “Nitrogen Dioxide”[tiab] OR “Nitrogen Peroxide”[tiab] OR “NO2”[tiab]	29732
#6	"Ozone"[Mesh] OR “Ozone”[tiab] OR “O3”[tiab]	43355
#5	"Sulfur Dioxide"[Mesh] OR “Sulfur Dioxide”[tiab] OR “Sulfurous Anhydride”[tiab] OR “SO2”[tiab]	19697
#4	"Particulate Matter"[Mesh] OR “Particulate Matter”[tiab] OR “Particle Pollutant*”[tiab] OR “Particulate Air Pollutant*”[tiab] OR “Ultrafine Particle*”[tiab] OR “Ultrafine Fiber*”[tiab] OR “PM10”[tiab] OR “PM 10”[tiab] OR “PM2.5”[tiab] OR “PM 2.5”[tiab]	107459

#3	"Air Pollutants"[Mesh] OR “Air Pollutant*”[tiab] OR “Air Environmental Pollutant*”[tiab]	92603
#2	"Air Pollution"[Mesh] OR “Air Pollution”[tiab]	96528
#1	"Alzheimer Disease"[Mesh] OR “Alzheimer’s Disease*”[tiab] OR “Alzheimer Disease*”[tiab] OR “Alzheimer’s Syndrome*”[tiab] OR “Alzheimer Syndrome*”[tiab] OR “Alzheimer-Type Dementia*”[tiab] OR “Alzheimer Type Dementia*”[tiab] OR “Alzheimer Dementia*”[tiab] OR “Alzheimer’s Dementia*”[tiab] OR “Senile Dementia”[tiab] OR “Primary Senile Degenerative Dementia”[tiab] OR “Alzheimer’s Sclerosis”[tiab] OR “Alzheimer Sclerosis”[tiab] OR “Presenile Dementia*”[tiab]	221613
