# Supplementary material for: Air Pollution and Alzheimer’s Disease: A Systematic Review and Meta-Analysis
Source: J Clin Med. 2026 May 28;15(11):4163. doi: 10.3390/jcm15114163 (PMC13257598; doi:10.3390/jcm15114163)
Supplement: Supplementary file 1 [file jcm-15-04163-s001.zip › Table S3.pdf]

Table S3. Embase (via OVID) Search History

Search	Embase (via OVID) Query – 18th May, 2025	Items found
16	14 NOT 15	490
15	limit 14 to conference abstract status	44
14	12 AND 13	534
13	4 OR 5 OR 6 OR 7 OR 8 OR 9 OR 10	1347193
12	1 AND 11	804
11	2 OR 3	238434
10	Smog/ OR “Smog”.ti,ab,kf.	3316
9	Exhaust gas/ OR “Vehicle Emission*”.ti,ab,kf. OR “Vehicular Emission*”.ti,ab,kf. OR “Diesel Exhaust”.ti,ab,kf. OR “Automobile xhaust”.ti,ab,kf. OR “Engine Exhaust”.ti,ab,kf. OR “Traffic-Related Pollutant*”.ti,ab,kf. OR “Transportation Emission*”.ti,ab,kf.	25449
8	Carbon monoxide/ OR “Carbon Monoxide”.ti,ab,kf. OR “CO”.ti,ab,kf.	1175924
7	Nitrogen dioxide/ OR “Nitrogen Dioxide”.ti,ab,kf. OR “Nitrogen Peroxide”.ti,ab,kf. OR “NO2”.ti,ab,kf.	40151
6	Ozone/ OR “Ozone”.ti,ab,kf. OR “O3”.ti,ab,kf.	55658
5	Sulfur dioxide/ OR “Sulfur Dioxide”.ti,ab,kf. OR “Sulfurous Anhydride”.ti,ab,kf. OR “SO2”.ti,ab,kf.	30191

4	Particulate matter/ OR “Particulate Matter”.ti,ab,kf. OR “Particle Pollutant*”.ti,ab,kf. OR “Particulate Air Pollutant*”.ti,ab,kf. OR “Ultrafine Particle*”.ti,ab,kf. OR “Ultrafine Fiber*”.ti,ab,kf. OR “PM10”.ti,ab,kf. OR “PM 10”.ti,ab,kf. OR “PM2.5”.ti,ab,kf. OR “PM 2.5”.ti,ab,kf.	89144
3	Air pollutant/ OR “Air Pollutant*”.ti,ab,kf. OR “Air Environmental Pollutant*”.ti,ab,kf.	123016
2	Air pollution/ OR “Air Pollution”.ti,ab,kf.	236243
1	Alzheimer disease/ OR “Alzheimer’s Disease*”.ti,ab,kf. OR “Alzheimer Disease*”.ti,ab,kf. OR “Alzheimer’s Syndrome*”.ti,ab,kf. OR “Alzheimer Syndrome*”.ti,ab,kf. OR “Alzheimer-Type Dementia*”.ti,ab,kf. OR “Alzheimer Type Dementia*”.ti,ab,kf. OR “Alzheimer Dementia*”.ti,ab,kf. OR “Alzheimer’s Dementia*”.ti,ab,kf. OR “Senile Dementia”.ti,ab,kf. OR “Primary Senile Degenerative Dementia”.ti,ab,kf. OR “Alzheimer’s Sclerosis”.ti,ab,kf. OR “Alzheimer Sclerosis”.ti,ab,kf. OR “Presenile Dementia*”.ti,ab,kf.	334167
