# Supplementary material for: Air Pollution and Alzheimer’s Disease: A Systematic Review and Meta-Analysis
Source: J Clin Med. 2026 May 28;15(11):4163. doi: 10.3390/jcm15114163 (PMC13257598; doi:10.3390/jcm15114163)
Supplement: Supplementary file 1 [file jcm-15-04163-s001.zip › Table S4.pdf]

Table S4. Scopus Search History

Search	Scopus Query – 18th May, 2025	Items found
14	12 AND 13	271
13	4 OR 5 OR 6 OR 7 OR 8 OR 9 OR 10	3123663
12	1 AND 11	397
11	2 OR 3	139948
10	TITLE-ABS(“Smog”)	7447
9	TITLE-ABS(“Vehicle Emission*”) OR TITLE-ABS(“Vehicular Emission*”) OR TITLE-ABS(“Diesel Exhaust”) OR TITLE-ABS(“Automobile Exhaust”) OR TITLE-ABS(“Engine Exhaust”) OR TITLE-ABS(“Traffic-Related Pollutant*”) OR TITLE-ABS(“Transportation Emission*”)	29258
8	TITLE-ABS(“Carbon Monoxide”) OR TITLE-ABS(“CO”)	2730152
7	TITLE-ABS(“Nitrogen Dioxide”) OR TITLE-ABS(“Nitrogen Peroxide”) OR TITLE-ABS(“NO2”)	93685
6	TITLE-ABS(“Ozone”) OR TITLE-ABS(“O3”)	174091
5	TITLE-ABS(“Sulfur Dioxide”) OR TITLE-ABS(“Sulfurous Anhydride”) OR TITLE-ABS(“SO2”)	90908
4	TITLE-ABS(“Particulate Matter”) OR TITLE-ABS(“Particle Pollutant*”) OR TITLE-ABS(“Particulate Air Pollutant*”) OR TITLE-ABS(“Ultrafine Particle*”) OR TITLE-ABS(“Ultrafine Fiber*”) OR TITLE-ABS(“PM10”)	129251

	OR TITLE-ABS(“PM 10”) OR TITLE-ABS(“PM2.5”) OR TITLE-ABS(“PM 2.5”)	
3	TITLE-ABS(“Air Pollutant*”) OR (“Air Environmental Pollutant*”)	43011
2	TITLE-ABS(“Air Pollution”)	114664
1	TITLE-ABS(“Alzheimer’s Disease*”) OR TITLE-ABS(“Alzheimer Disease*”) OR TITLE-ABS(“Alzheimer’s Syndrome*”) OR TITLE-ABS(“Alzheimer Syndrome*”) OR TITLE-ABS(“Alzheimer-Type Dementia*”) OR TITLE-ABS(“Alzheimer Type Dementia*”) OR TITLE-ABS(“Alzheimer Dementia*”) OR TITLE-ABS(“Alzheimer’s Dementia*”) OR TITLE-ABS(“Senile Dementia”) OR TITLE-ABS(“Primary Senile Degenerative Dementia”) OR TITLE-ABS(“Alzheimer’s Sclerosis”) OR TITLE-ABS(“Alzheimer Sclerosis”) OR TITLE-ABS(“Presenile Dementia*”)	232025
