# Supplementary material for: Air Pollution and Alzheimer’s Disease: A Systematic Review and Meta-Analysis
Source: J Clin Med. 2026 May 28;15(11):4163. doi: 10.3390/jcm15114163 (PMC13257598; doi:10.3390/jcm15114163)
Supplement: Supplementary file 1 [file jcm-15-04163-s001.zip › Table S5.pdf]

Table S5. Characteristics of excluded studies

Article	Reasons for exclusions
Guo 2025 [43]	Study not limited to AD
Thompson 2025 [44]	Study not limited to AD
Casey 2024 [45]	Hazard ratio missed
Zhang 2024 [46]	Study not limited to AD
Ji 2024 [47]	Pre-print
Blanco 2024 [48]	Study not limited to AD<br>HR missing
Ge 2024 [49]	Not specific outcomes for particulate
Gialluisi 2023 [50]	Pre-print
Semmens 2023 [51]	Study not regarding AD
De Crom 2023 [52]	Not exploring the influence of air pollution on AD
Andersson 2023 [53]	Investigation about olfaction
Petkus 2023 [54]	Pre-print
Bishop 2023 [55]	Study not limited to AD
Lee 2023 [56]	Not exploring the influence of air pollution on AD
Rodriguez-Loureiro 2022 [57]	Study not regarding air pollution
Wang 2022 [58]	Study not limited to AD
Åström 2021 [59]	Commentary
Alemany 2021 [60]	No AD Risk
Sullivan 2021 [61]	Study not limited to AD
Younan 2021 [62]	Study focused on older woman
Crous-Bou 2020 [63]	Not exploring the influence of air pollution on AD
Grande 2020 [64]	Study not limited to AD
Lee 2020 [65]	Study regarding beta-amyloid burden
Paul 2020 [66]	Study not limited to AD
Younan 2020 [67]	Inadequate statistical analysis
Li 2019 [68]	Study about rhinitis

Chen 2017 [69]	Study not limited to AD
AD: Alzheimer’s disease; HR: hazard ratio	
