# Supplementary material for: Air Pollution and Alzheimer’s Disease: A Systematic Review and Meta-Analysis
Source: J Clin Med. 2026 May 28;15(11):4163. doi: 10.3390/jcm15114163 (PMC13257598; doi:10.3390/jcm15114163)
Supplement: Supplementary file 1 [file jcm-15-04163-s001.zip › Table S6.pdf]

Table S6. Quality appraisal for included articles

Study	Showed the data source (such as the Center for Medicaid and Medicare)	Showed the study design (such as case control)	Provided the criteria of inclusion and exclusion for participants selection	Provided the age information of population (such as mean age)	Indicated the demographics of subjects (e.g., an elderly population from northeastern United States)	Provided the population size	Showed the implement for the quality assurance purpose (such as sensitivity analysis)	Described the exclusion criteria of specified data in the study	Adjusted the confounding or other effects from the results (such as the adjustment of age)	Described whether there was any missing data and how it was handled	Total score
Qin 2025 [11]	1	1	0	1	1	1	1	0	1	1	8
Zheng 2025 [20]	1	1	1	1	1	1	1	1	1	1	10
Zhu 2025 [21]	1	1	1	0	0	1	1	0	1	1	7
Tian 2024 [22]	1	1	1	1	1	1	1	1	1	1	10
Zhang 2024 [23]	1	1	1	1	1	1	1	1	1	1	10
Ma 2023 [10]	1	1	1	1	1	1	1	1	1	1	10
Yuan 2023 [24]	1	1	1	0	1	1	1	1	1	1	9
Zhu 2023 [25]	1	1	1	1	1	1	1	1	1	1	10
Parra 2022 [26]	1	1	1	0	1	1	1	0	1	1	8
Shi 2022 [27]	1	1	1	1	1	1	1	1	1	1	10
Yang 2022 [28]	1	1	1	1	1	1	1	1	1	1	10
Zhang 2022 [2]	1	1	1	1	1	1	1	1	1	1	10
Mortamais 2021 [29]	1	1	1	1	1	1	1	1	1	1	10
Ran 2021 [30]	1	1	1	1	1	1	1	1	1	1	10

Rhew 2021 [31]	1	1	1	0	1	1	1	0	0	0	6
Shi 2021 [32]	1	1	1	1	1	1	1	1	1	1	10
Shi 2020 [33]	1	1	1	1	1	1	1	0	1	1	9
Smargiassi 2020 [34]	1	1	1	1	1	1	1	1	1	1	10
Yuchi 2020 [35]	1	1	1	1	1	1	1	1	1	1	10
Cerza 2019 [36]	1	1	1	1	1	1	1	1	1	1	10
Carey 2018 [37]	1	1	1	1	1	1	1	1	1	1	10
Oudin 2018 [38]	1	1	1	1	1	1	1	1	1	1	10
Culqui 2017 [39]	1	1	1	1	1	1	1	1	1	1	10
Kioumourtzoglou 2016 [40]	1	1	1	1	1	1	1	1	1	1	10
Oudin 2016 [41]	1	1	1	0	1	1	1	1	1	1	9
Jung 2015 [42]	1	1	1	1	1	0	1	1	1	0	8
Wu 2015 [1]	1	1	1	1	1	1	1	1	1	1	10
