# Supplementary figures and images for: Air Pollution and Alzheimer’s Disease: A Systematic Review and Meta-Analysis
Source: J Clin Med. 2026 May 28;15(11):4163. doi: 10.3390/jcm15114163 (PMC13257598; doi:10.3390/jcm15114163)

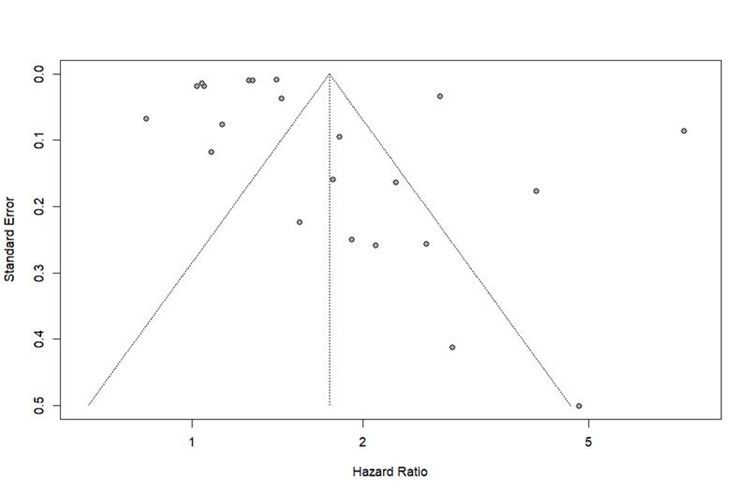

Supplement: Supplementary file 1 [file jcm-15-04163-s001.zip › Figure S1.jpg]

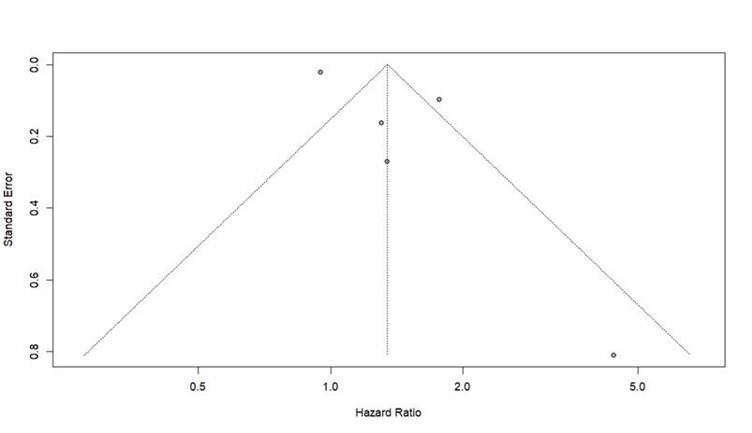

Supplement: Supplementary file 1 [file jcm-15-04163-s001.zip › Figure S2.jpg]

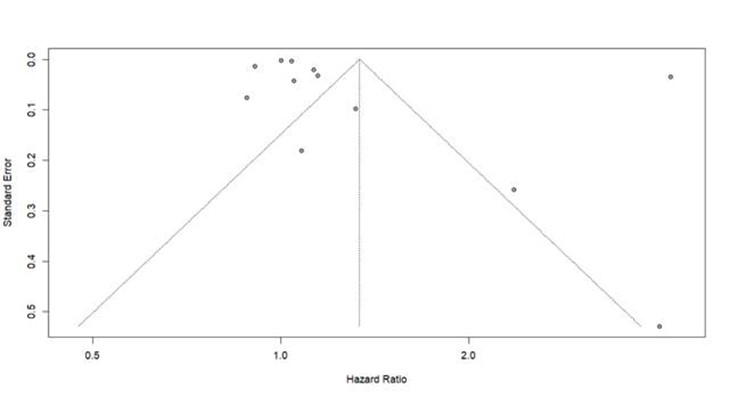

Supplement: Supplementary file 1 [file jcm-15-04163-s001.zip › Figure S3.jpg]

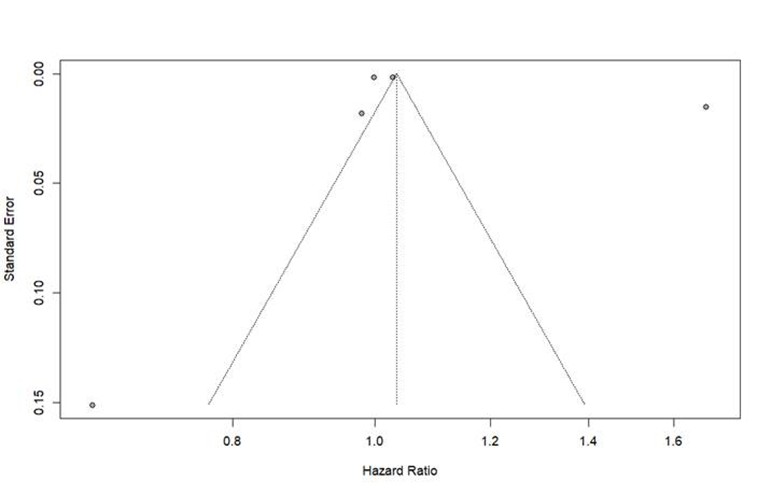

Supplement: Supplementary file 1 [file jcm-15-04163-s001.zip › Figure S4.jpg]

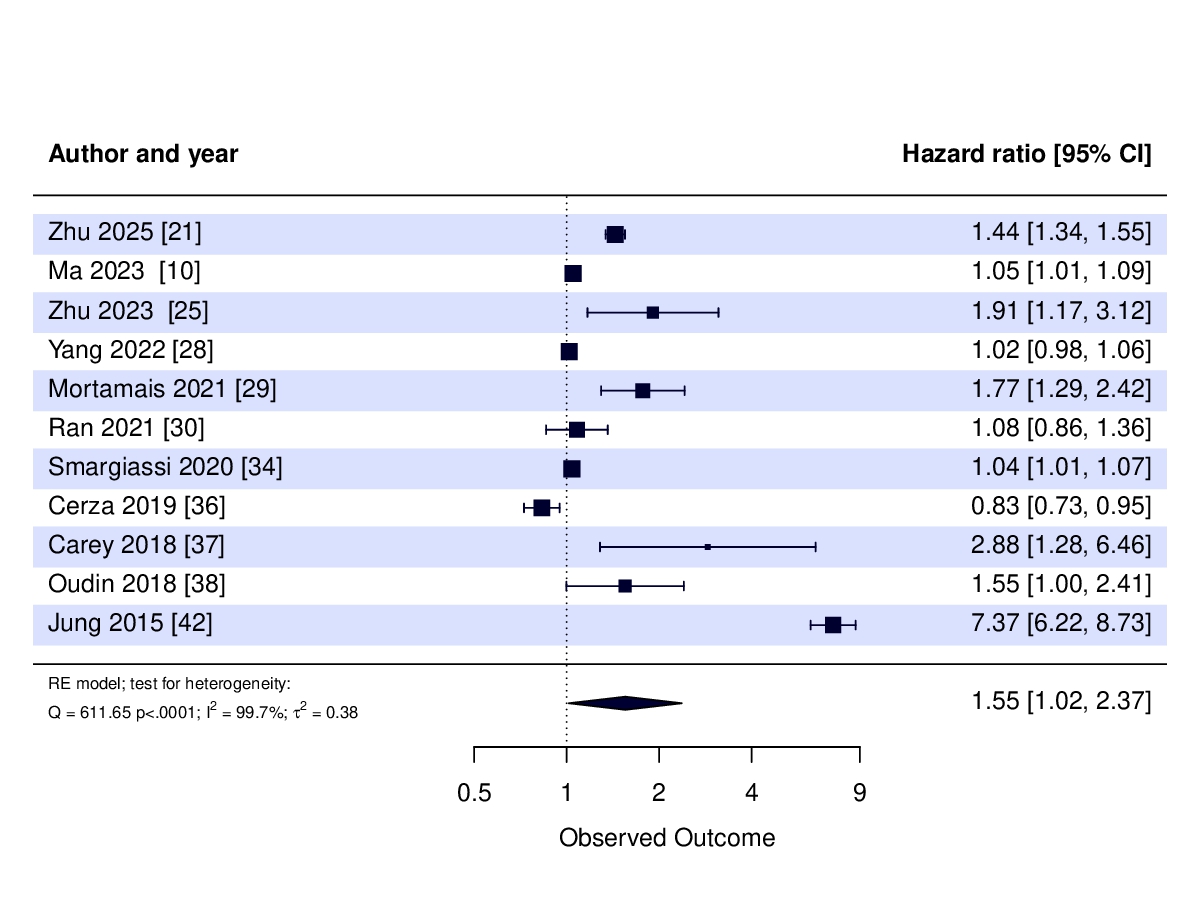

Supplement: Supplementary file 1 [file jcm-15-04163-s001.zip › Figure S5.jpg]

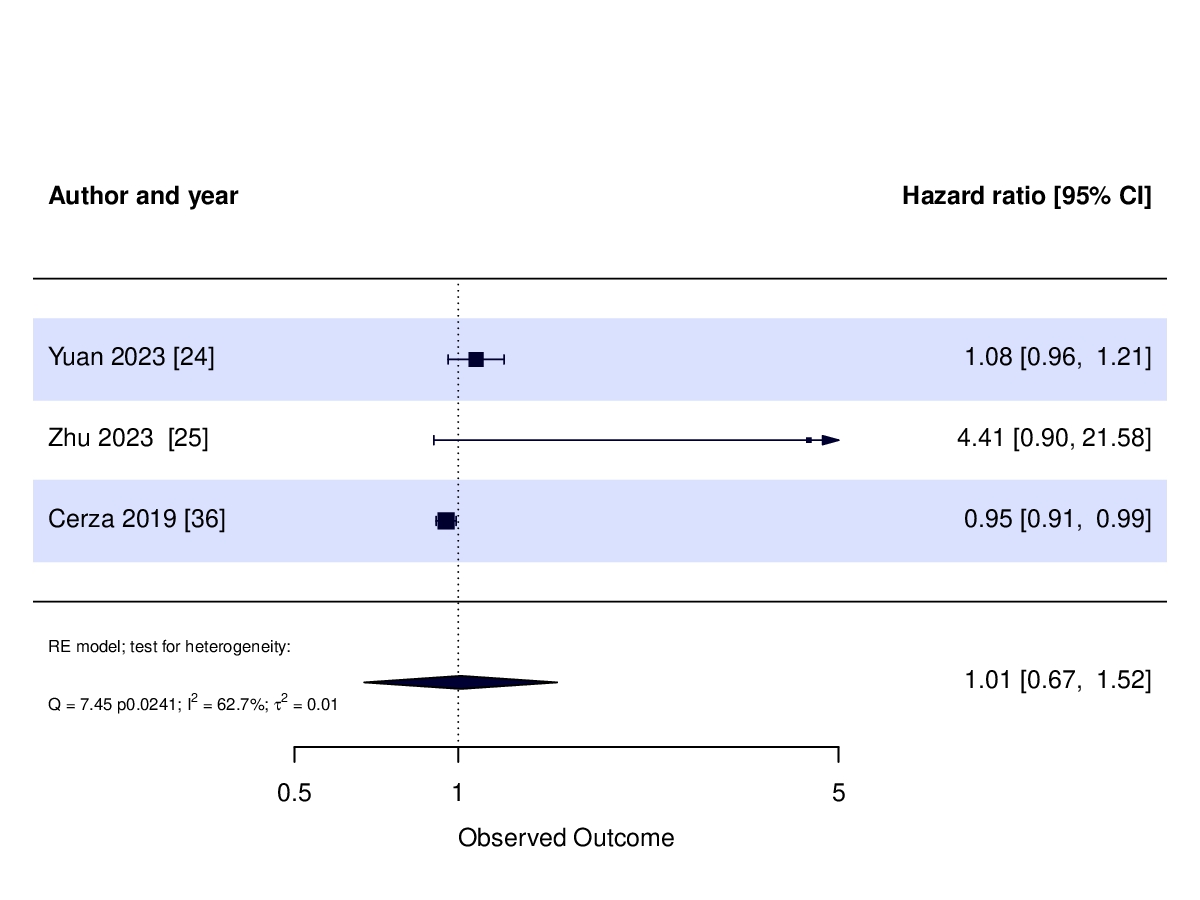

Supplement: Supplementary file 1 [file jcm-15-04163-s001.zip › Figure S6.jpg]

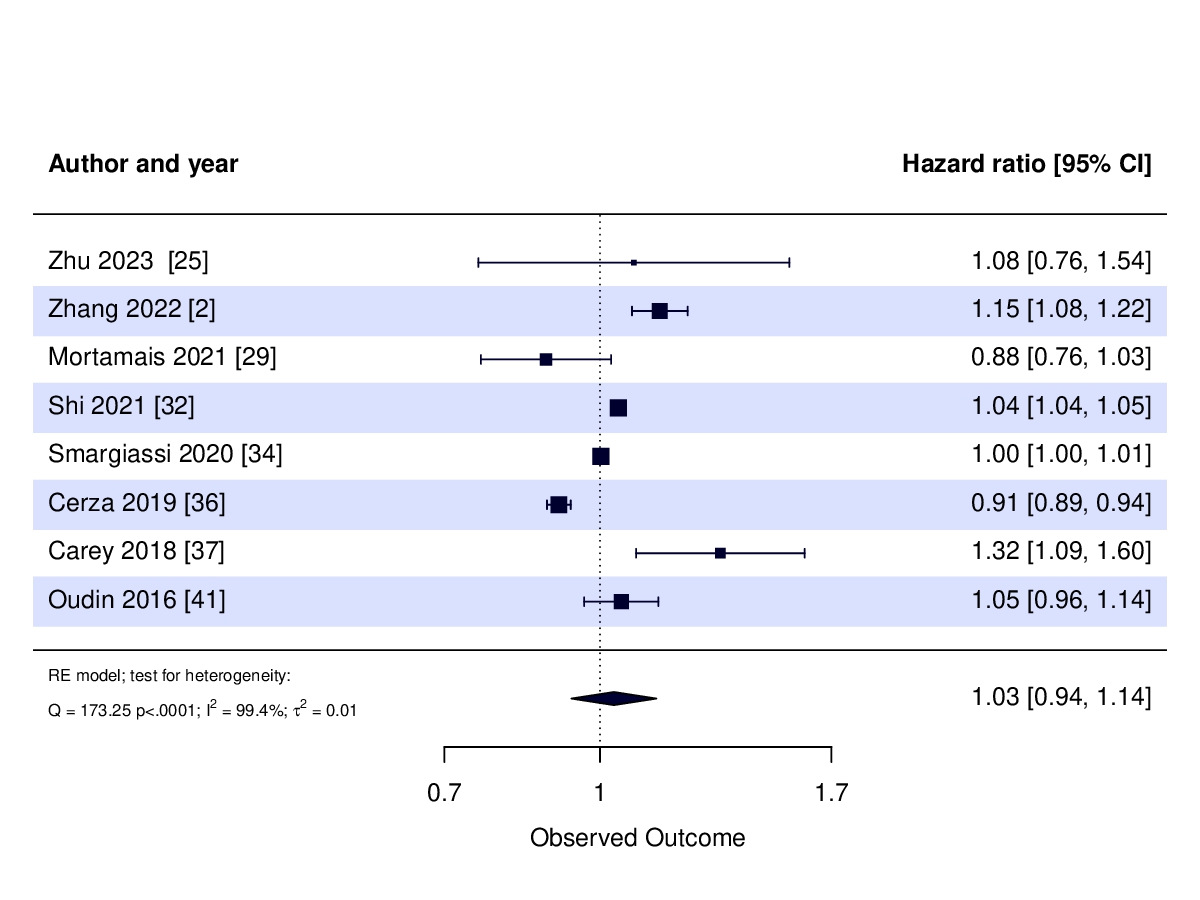

Supplement: Supplementary file 1 [file jcm-15-04163-s001.zip › Figure S7.jpg]

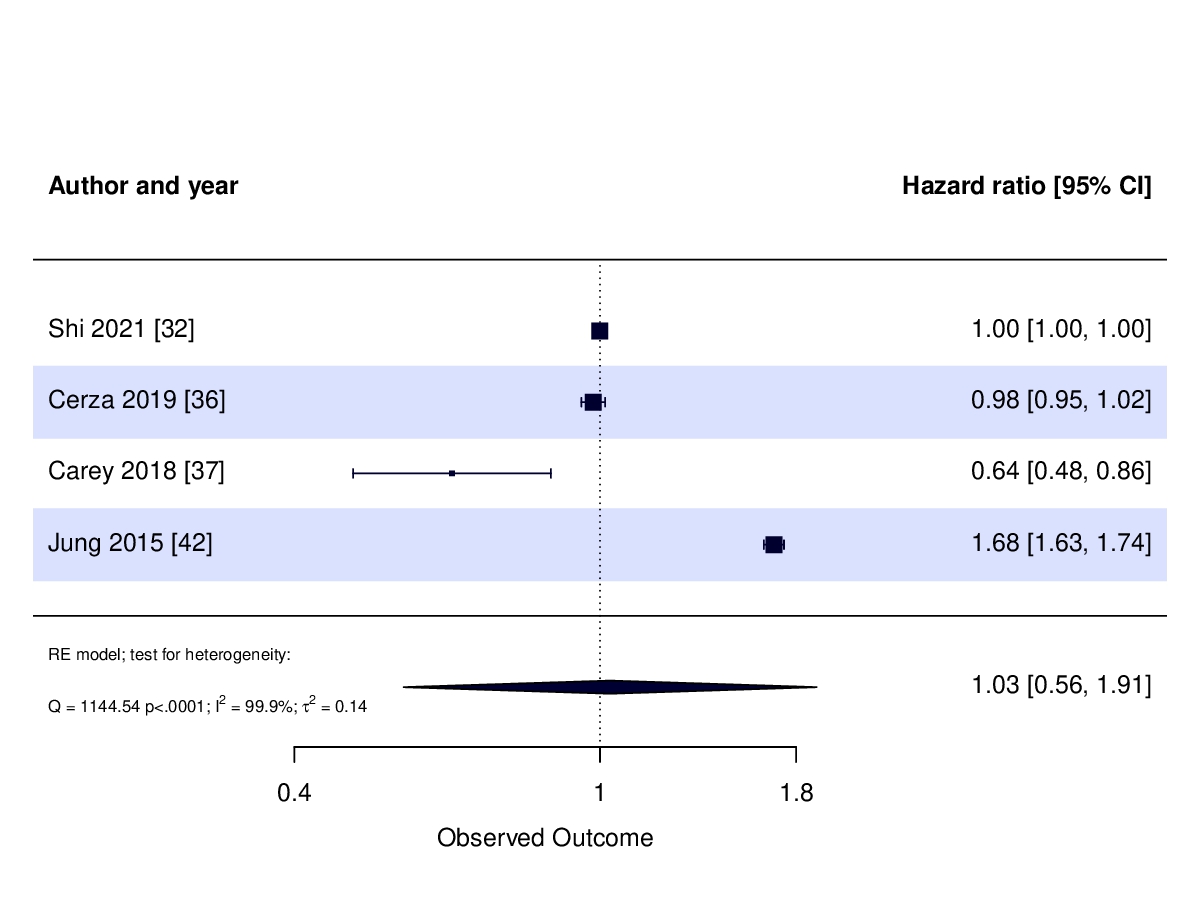

Supplement: Supplementary file 1 [file jcm-15-04163-s001.zip › Figure S8.jpg]
